# Cost-effectiveness of interventions for medically unexplained symptoms: A systematic review

**DOI:** 10.1371/journal.pone.0205278

**Published:** 2018-10-15

**Authors:** Margreet S. H. Wortman, Joran Lokkerbol, Johannes C. van der Wouden, Bart Visser, Henriëtte E. van der Horst, Tim C. olde Hartman

**Affiliations:** 1 ACHIEVE – Centre of Applied Research, Faculty of Health, Amsterdam University of Applied Sciences, Amsterdam, The Netherlands; 2 Department of General Practice and Elderly Care Medicine, Amsterdam UMC, Vrije Universiteit, Amsterdam, The Netherlands; 3 Centre of Economic Evaluation, Trimbos Institute (Netherlands Institute of Mental Health and Addiction), Utrecht, The Netherlands; 4 Department of Health Care Policy, Harvard Medical School, Boston, Massachusetts, United States of America; 5 Department of Primary and Community Care, Radboud University Medical Center, Nijmegen, The Netherlands; Universitat Bremen, GERMANY

## Abstract

**Background:**

In primary and secondary care medically unexplained symptoms (MUS) or functional somatic syndromes (FSS) constitute a major burden for patients and society with high healthcare costs and societal costs. Objectives were to provide an overview of the evidence regarding the cost-effectiveness of interventions for MUS or FSS, and to assess the quality of these studies.

**Methods:**

We searched the databases PubMed, PsycINFO, the National Health Service Economic Evaluation Database (NHS-EED) and the CEA registry to conduct a systematic review. Articles with full economic evaluations on interventions focusing on adult patients with undifferentiated MUS or fibromyalgia (FM), irritable bowel syndrome (IBS) and chronic fatigue syndrome (CFS), with no restrictions on comparators, published until 15 June 2018, were included. We excluded preventive interventions. Two reviewers independently extracted study characteristics and cost-effectiveness data and used the Consensus on Health Economic Criteria Checklist to appraise the methodological quality.

**Results:**

A total of 39 studies out of 1,613 articles met the inclusion criteria. Twenty-two studies reported costs per quality-adjusted life year (QALY) gained and cost-utility analyses (CUAs). In 13 CUAs the intervention conditions dominated the control conditions or had an incremental cost-effectiveness ratio below the willingness-to-pay threshold of € 50,000 per QALY, meaning that the interventions were (on average) cost-effective in comparison with the control condition. Group interventions focusing on MUS (*n* = 3) or FM (*n* = 4) might be more cost-effective than individual interventions. The included studies were heterogeneous with regard to the included patients, interventions, study design, and outcomes.

**Conclusion:**

This review provides an overview of 39 included studies of interventions for patients with MUS and FSS and the methodological quality of these studies. Considering the limited comparability due to the heterogeneity of the studies, group interventions might be more cost-effective than individual interventions.

**Registration:**

Study methods were documented in an international prospective register of systematic reviews (PROSPERO) protocol, registration number: CRD42017060424.

## Introduction

Patients with medically unexplained symptoms (MUS, i.e. physical symptoms for which no pathological cause can be found after adequate physical examination) are highly prevalent in primary and secondary care in all medical settings [1,2]. The classification of these physical symptoms is problematic as numerous overlapping diagnoses and syndrome labels show [3]. Almost each medical specialty has defined its own syndrome(s) based on symptoms that relate to their organ of interest [4]. Psychiatry uses the designation somatic symptom disorder, while most medical specialties have patients with clusters of MUS within so called ‘functional somatic syndromes’ (FSS) [1] e.g. fibromyalgia (FM) [5], irritable bowel syndrome (IBS) [6], chronic fatigue syndrome (CFS) [7], chronic benign pain syndrome and multiple chemical sensitivity (MCS) [4,8]. The most well-known FSS are FM, IBS, and CFS [9] and most primary care physicians and researchers are familiar with the umbrella term MUS [10].

MUS are often accompanied by psychological distress, social isolation and reduced quality of life [11,12]. Severe MUS are associated with multiple functional impairments and psychiatric morbidity [13–15]. Patients with MUS and FSS suffer from their symptoms, are functionally impaired [9] and are at risk for false-positive diagnostic tests, potentially harmful additional testing and treatment procedures [16]. Therefore, these symptoms constitute a major burden on patients and society with considerable societal costs, health care costs and costs of lost productivity [9]. In a Dutch study (2005–2008) the mean total cost, both the use of healthcare services (direct costs) and productivity-related costs (indirect costs), was estimated to be € 6,815 per patient per year [12]. In a German study (2007–2009), outpatient physician visits were the most expensive single cost category of the direct costs and indirect costs were predominantly caused by productivity reduction at work [17].

Little is known about the cost-effectiveness and methodological quality of economic evaluations of interventions for patients with MUS and FSS. Although helpful for policy makers, systematic reviews of cost-effectiveness data in this area are scarce. Earlier, Konnopka et al. [18] published a systematic review of health economics studies for MUS. The aim of that systematic review was to give an overview of both cost-of-illness studies and economic evaluations for patients with MUS. Since in the review by Konnopka et al. [18] the quality of the included studies was not addressed and the included studies were only up to 2008, we consider an update is due.

Therefore, the objectives of this review are to provide an overview of the evidence regarding the cost-effectiveness of interventions for patients with MUS and FSS, and to assess the methodological quality of the identified economic evaluations.

## Methods

The methods and reporting of this systematic review are in concordance with the Preferred Reporting Items for Systematic Reviews and Meta-Analyses (PRISMA) guidelines (S1 Table) [19]. Prior to the start of article inclusion, we documented study methods in an international prospective register of systematic reviews (PROSPERO) protocol (S1 Text), registration number CRD42017060424.

### Literature search and study selection

We performed a literature search until 15 June 2018 in the following databases: PubMed, PsycINFO, the National Health Service Economic Evaluation Database (NHS-EED), and the CEA registry. The NHS-EED is a health economic database including economic evaluations. The CEA registry includes studies in which a cost-effectiveness analysis was performed. In addition to free-text terms, we used Medical Subject Headings (MeSH) terms and Psychological Index Terms for searches within the PubMed and PsycINFO databases, respectively. In order to identify economic evaluations on MUS, we selected key terms that were used in a Cochrane review on non-pharmacological interventions for somatoform disorders and medically unexplained physical symptoms (MUPS) in adults [20] and combined these with health-economic key terms. An information specialist was involved in the development of the search strategy. A detailed description of the search strategy for every database can be found in the supplementary files (S1 Appendix). Additionally, we checked existing systematic reviews and the references of studies included in our review manually for relevant studies.

We included studies on adult patients with MUS, reporting on psychological, physical/exercise, internet-delivered, pharmacological and combined interventions compared with usual care, waiting list, other physical or psychological treatment and describing health care use or societal costs.

Only studies reporting on full economic evaluations were included, meaning that the studies compared both costs and effects of two or more conditions [21]. We excluded studies when interventions focused on prevention or screening. We limited the scope of the studies to adult patients with undifferentiated MUS and the three most common specific functional syndromes FM, IBS and CFS. We excluded studies with medically (partly) explained symptoms or medically unexplained symptoms as secondary diagnosis. The literature search was limited to publications written in English, Dutch and German. We included both trial-based economic evaluations (TBEEs) and model-based economic evaluations (MBEEs). In TBEEs costs and effects are measured alongside an effectiveness trial, whereas in MBEEs available evidence is synthesized and used to simulate (often long term) effectiveness and costs. We excluded study protocols and included only original research.

Titles and abstracts of the search results were independently screened by two reviewers (MSHW and JL). Studies that were in agreement with the inclusion criteria based on title and abstract were retrieved as full text. Disagreements about the eligibility of studies were resolved in a consensus meeting. A third reviewer (BV) was available in case of disagreement.

The full text articles were evaluated independently by the two reviewers (MSHW and JL) to assess eligibility. In a consensus meeting the full text articles were discussed and discrepancies between the two researchers were resolved by consensus, and when needed a third researcher (BV) was consulted.

### Data extraction

Two reviewers (MSHW and JL) independently extracted data and assessed the methodological quality of each study. The articles excluded on full-text level were documented and are provided in the supplementary files (S2 Table). The development of the data extraction form was based on a previous review by one of the authors (JL) [22]. To pilot this data extraction form, the reviewers screened the first eight articles together. After adaptation of the draft extraction form, we extracted from each of the included articles the following information: name first author, country, study design economic evaluation, target population, perspective, time horizon, treatment alternatives (intervention, comparators and sample size), effect measurement and valuation, discount rates, valuation year, costs categories, incremental costs, incremental effects and health economic results.

### Quality assessment

The methodological quality of the studies was assessed with the extended Consensus on Health Economic Criteria (CHEC) list [23], which is recommended by the Cochrane Handbook for Systematic Reviews of Interventions [24] for critical appraisal of the methodological quality of health-economic evidence. The checklist contains 20 items covering the quality of the design and reporting of the economic evaluation studies. Although the CHEC is not optimal for assessing the methodological quality of MBEEs, we chose the CHEC for the quality assessment of both TBEEs and MBEEs in order to optimize comparability of the results. Each question on the CHEC checklist was scored with either ‘Yes’ (score 1), ‘Suboptimal’ (score 0.5), ‘No’ (score 0), ‘NA’ (not applicable) or ‘Uncertain’ (no score). The ‘Uncertain’ option was used only when information on an item was not entirely clear. We did not contact authors when the published information was insufficient to assign a score.

Prior to the quality assessment, to improve uniform scoring, two reviewers (MSHW and JL) independently assessed and discussed eight included studies (two of each target population: MUS, FM, IBS and CFS). A detailed description of the scoring instructions is provided in the supplementary files (S2 Appendix). Two reviewers (MSHW and JL) assessed the quality of each study independently. Disagreements between the two reviewers were resolved in a consensus meeting.

### Outcomes

For each study, we extracted the incremental costs, incremental effects and incremental cost-effectiveness ratio (ICER), indicating the costs per additional quality-adjusted life year (QALY) or any other (clinical) outcome. To enhance comparability of the health economic results between studies conducted in different countries and at different years, ICERs were converted to 2016 Euro using Purchasing Power Parity (PPP) rates [25] and the Consumer Price Index [26]. To assess cost-effectiveness for studies reporting the cost per additional QALY, one year in perfect health, we applied an overall willingness-to-pay (WTP) threshold of € 50,000 per QALY, a commonly used threshold in the Netherlands [27]. WTP thresholds are not available for other outcome measures. The WTP threshold refers to the maximum amount a country or society is willing to pay for a particular health gain [28]. When an ICER is below the WTP threshold, the intervention can be regarded as on average cost-effective in comparison with the comparator. In accordance with the Consolidated Health Economic Evaluation Reporting Standards (CHEERS), we distinguished healthcare and societal economic perspectives [29]. Due to heterogeneity, a meta-analysis could not be conducted.

## Results

### Literature search and study selection

**Fig 1:**
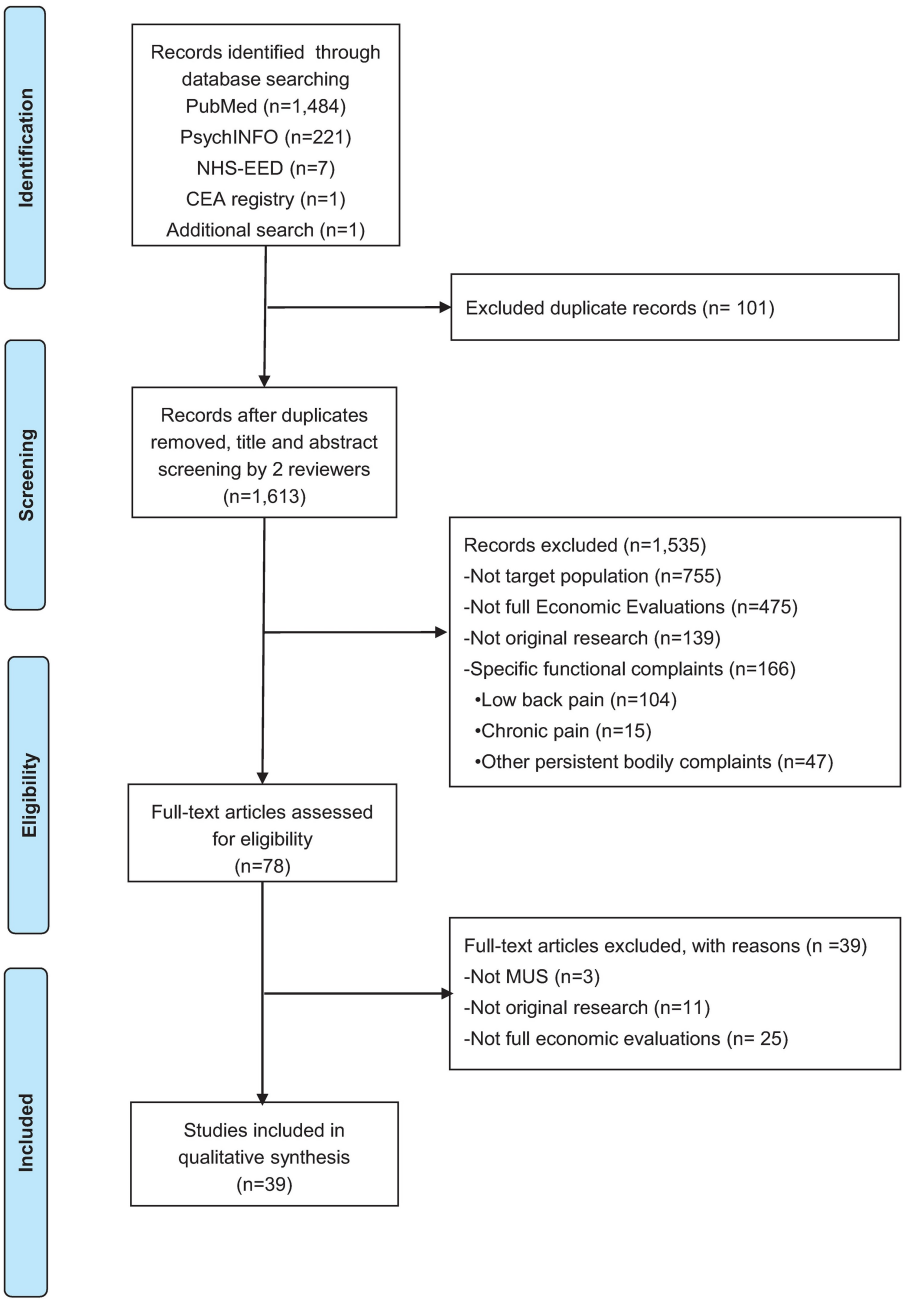
Flow diagram of the study identification process.

In total, the search strategy yielded 1,713 articles. One study was found by additional reference searching. After excluding 101 duplicates, the titles and abstracts of 1,613 articles were screened for relevance. Title and abstract screening resulted in the exclusion of 1,535 articles, mainly because they were not (full) economic evaluations or not primarily focused on MUS. Of the 78 articles that were assessed full-text, 39 were excluded for being not full-economic evaluations (*n* = 25), not primarily focused on MUS or FSS (*n* = 3), or not being original research (*n* = 11). Finally, 39 articles were included for analysis. A flow diagram of the study identification process is presented in Fig 1.

### Data extraction

#### Overview of the included studies

The main characteristics of the included studies are presented in Table 1. The most recent study was published in July 2017 [30], and the oldest study was published in 1992 [31]. Most studies were conducted in Europe (*n* = 30): UK (*n* = 11), the Netherlands (*n* = 6), Germany (*n* = 3), Spain (*n* = 4), Sweden (*n* = 2), Denmark (*n* = 2), Scotland (*n* = 1) and Norway, Sweden, Finland, Denmark (*n* = 1). The remaining studies (*n* = 9) were conducted in the USA (*n* = 7), Mexico (*n* = 1) and one study had a global scope.

**Table 1:** Main characteristics of economic evaluations of interventions for MUS.

ID	Authors (year)	Country	Economic evaluation	Target population	analysis	Perspective	Time horizon	Industry funding
1	Schröder et al, 2017 [46]	Denmark	TBEE	MUS	CUA/CMA	Healthcare and societal	16 months	No
2	Konnopka et al, 2016 [54]	Germany	TBEE	MUS	CUA	Societal	1 year	No
3	Visser et al, 2015 [48]	Netherlands	MBEE	MUS	CUA	Healthcare and societal	4 years	No
4	Chernyak et al, 2014 [55]	Germany	TBEE	MUS	CUA	Healthcare	1 year	No
5	Van Ravesteijn et al, 2013 [56]	Netherlands	TBEE	MUS	CUA	Healthcare and societal	1 year	No
6	Barsky et al, 2013 [57]	United States	TBEE	MUS	CEA	Healthcare	1 year	No
7	Hiller et al, 2003 [41]	Germany	TBEE	MUS	CEA	Societal	2 years	No
8	Morriss et al, 1998 [33]	United Kingdom	TBEE	MUS	CEA	Healthcare	3 months	No
9	Smith et al, 1995 [32]	United States	TBEE	MUS	CEA	Not mentioned—Healthcare	2 years	No
10	Kashner et al, 1992 [31]	United States	TBEE	MUS	CEA	Healthcare	1 year	No
11	Luciano et al, 2017 [30]	Spain	TBEE	FM	CUA	Healthcare and societal	6 months	No
12	Luciano et al, 2014 [36]	Spain	TBEE	FM	CUA	Healthcare and societal	6 months	No
13	Luciano et al, 2013 [58]	Spain	TBEE	FM	CUA	Healthcare and societal	1 year	No
14	Arreola Ornelas et al, 2012 [52]	Mexico	MBEE	FM	CEA	Healthcare	1 year	Yes
15	Lloyd et al, 2012 [53]	United States	MBEE	FM	CEA	Societal	1 year	Yes
16	Beard et al, 2011 [42]	United States	MBEE	FM	CEA/CUA	Healthcare and societal	2 years	Yes
17	Choy et al, 2010 [47]	United Kingdom	MBEE	FM	CEA/CUA	Healthcare	3 years	Yes
18	Gusi et al, 2008 [37]	Spain	TBEE	FM	CUA	Healthcare and societal	8 months	No
19	Zijlstra et al, 2007 [59]	Netherlands	TBEE	FM	CUA	Societal	1 year	No
20	Goossens et al, 1996 [60]	Netherlands	TBEE	FM	CUA	Societal	1 year	No
21	Fisher et al, 2016 [49]	Scotland	MBEE	IBS	CUA	Healthcare	5 years	Yes
22	Tipsmark et al, 2016 [51]	Denmark	MBEE	IBS	CUA	Healthcare	20 years	No
23	Huang et al, 2015 [34]	United States	MBEE	IBS	CEA/CUA	Societal	12 weeks	Yes
24	Stamuli et al, 2012 [61]	United Kingdom	TBEE	IBS	CUA	Healthcare (NHS)	1 year	No
25	Andersson et al, 2011 [62]	Sweden	TBEE	IBS	CEA	Societal	12 months	No
26	Ljotsson et al, 2011 [63]	Sweden	TBEE	IBS	CEA	Societal	1 year	No
27	McCrone et al, 2008 [64]	United Kingdom	TBEE	IBS	CEA	Societal	1 year	No
28	Bracco et al, 2007 [35]	Norway, Sweden, Finland, Denmark	TBEE	IBS	CUA	Healthcare	12 weeks	Yes
29	Robinson et al, 2006 [65]	United Kingdom	TBEE	IBS	CEA	Healthcare	1 year	No
30	Spiegel et al, 2004 [50]	Global scope	MBEE	IBS	CEA	Healthcare	10 years	No
31	Creed et al, 2003 [43]	United Kingdom	TBEE	IBS	CEA	Societal	15 months	Unclear
32	Vos-Vromans et al, 2017 [66]	Netherlands	TBEE	CFS	CEA/CUA	Societal	1 year	No
33	Meng et al, 2014 [67]	United States	TBEE	CFS	CEA	Societal	1 year	No
34	Richardson et al, 2013 [44]	United Kingdom	TBEE	CFS	CUA	Healthcare	70 weeks	No
35	McCrone et al, 2012 [68]	United Kingdom	TBEE	CFS	CEA/CUA	Healthcare and societal	1 year	No
36	Sabes-Figuera et al, 2012 [38]	United Kingdom	TBEE	CFS	CEA	Healthcare	6 months	No
37	Severens et al, 2004 [45]	Netherlands	TBEE	CFS	CEA/CUA	Healthcare	14 months	No
38	McCrone et al, 2004 [39]	United Kingdom	TBEE	CFS	CEA	Societal	8 months	No
39	Chisholm et al, 2001 [40]	United Kingdom	TBEE	CFS	CEA	Societal	6 months	No

CEA: cost-effectiveness analysis, CUA: cost-utility analysis, MBEE: model-based economic evaluation, TBEE: trial-based economic evaluation, MUS: Medically Unexplained Symptoms, FM: Fibromyalgia, IBS: Irritable Bowel Syndrome, CFS: Chronic Fatigue Syndrome.

The majority (*n* = 30) of the studies were trial-based economic evaluations (TBEEs) of which 24 originated from randomized trials. Nine economic evaluations were based on economic modelling (MBEE).

In the included studies a societal perspective (*n* = 14) or a healthcare perspective (*n* = 15) or both perspectives (*n* = 9) were used. One study did not explicitly report the study perspective [32].

The time horizon was shorter than six months (*n* = 3) [33–35], between six months and eight months (*n* = 6) [30, 36–40], between 14 months and two years (*n* = 7) [32, 41–46] or three to 20 years (*n* = 5) [47–51]. The remaining studies had time horizons of one year (*n* = 18) [31, 52–68].

Seven studies [34,35,42,47,49,52,53] reported funding by a pharmaceutical company.

Data on study population and treatment alternatives are presented in Table 2. Studies focused on patients with undifferentiated MUS (*n* = 10) [31–33,41,46,48,54–57], on patients with FM (*n* = 10) [30,36,37,42,47,52,53,58–60], on patients with IBS (*n* = 11) [34,35,43,49,50,51,61–65], and on patients with CFS (*n* = 8) [38–40,44,45,66–68].

**Table 2:** Characteristics of and results for economic evaluations of interventions for MUS.

ID (ref)	Target population	Treatment alternatives (*n*)^a^	Effect measurement and valuation^b^	Discount rates	Valuation year	Costs categories	Incremental costs [95%CI]*(treatment I vs treatment II and for all costs unless stated otherwise)	Incremental effects [95%CI]*(treatment I vs treatment II and for all costs unless stated otherwise)	Health economic results*
1 [46]	Patients (20–45) with multiple functional somatic symptoms for at least 2 years within a general hospital setting, referred by their primary care physician	I: Specialised Treatment for Severe Bodily Distress Syndromes (group CBT program (STreSS)) (54); II enhanced usual care (66)	QALYs (SF-6D) and self-rated physical health	No discounting applied	2010	Healthcare costs, indirect costs and public expenses associated with occupational status and social benefits	Healthcare perspective: -€1,004 [€-4,128; €2,120] Societal perspective: €940 [€−5,551; €7,432]	QALY: 0.035 [0.00; 0.07] Self-rated physical health: 20% [0.4%; 39%]	Healthcare perspective: STreSS was on average dominant for both outcomes. Societal perspective: The ICERs were €26,988 per QALY and €4,817 per patient improved.
2 [54]	Patients with functional somatic syndromes	I: Collaborative group intervention (CGI) (183); II: enhanced medical care (EMC) (145)	QALY (SF-6D)	NA	2007	Healthcare costs and productivity losses	Societal perspective: -€1,244. [CI NR]	QALY: 0.017 [CI NR]	On average, CGI dominated EMC.
3 [48]	Patients with a diagnosis of unexplained physical symptoms according to DSM-IV criteria	I: Cognitive behavioural group training (CBGT) (84); II: wait-list (WL) (78)	QALYs (SF-36)	4% costs; 1.5% effects	2011	Healthcare costs and work related costs	Healthcare perspective: €513 [CI NR] Societal perspective: -€886 [CI NR]	QALY: 0.06 [CI NR]	Healthcare perspective: ICER: €8,738 per QALY Societal perspective: The group training was dominant on average.
4 [55]	Patients with multisomatoform disorder	I: Psychodynamic interpersonal therapy (PIT) (106); II: enhanced medical care (EMC) (102)	QALY (SF-6D)	NA	NR	Treatment costs	Healthcare perspective: €784 [CI NR]	QALY: 0.02 [-0.01; 0.05]	Healthcare perspective: After multiple imputation, the ICER was €46,194 per QALY.
5 [56]	Patients belonging to the 10% most frequently attending patients in the participating GPs, fulfilling the DSM-IV criteria of an undifferentiated somatoform disorder	I: Mindfulness-based cognitive therapy (64); II: Enhanced usual care (61)	QALYs (SF-6D)	NA	2010	Healthcare costs and productivity losses	Healthcare perspective: €828 [CI NR] Societal perspective: €714; [€-1,726; €3,237]	QALY: 0.012. [-0.019; 0.041]	Healthcare perspective: ICER: €72,782 per QALY Societal perspective: ICER: €62,034 per QALY.
6 [57]	The highest 20% outpatient utilizers	I: Two-step cognitive behavioural therapy accompanied by a training seminar for their primary care physicians (CBT) (59); II: relaxation training (RT) (30)	Hypochondriasis (Whiteley score);	NA	NR	Healthcare costs	Healthcare perspective: Not reported for two conditions separately. For both groups combined, there is an average cost reduction of €522 in the year preceding versus the year following the interventions.	Whiteley score not reported separately for both conditions.	Healthcare perspective: ICER not reported
7 [41]	Patients with medically unexplained somatic symptoms in a German tertiary care facility	I: Cognitive Behavioural treatment program (SFD group)(172); II: regular treatment program (123)	SOMS; WI; CABAH; BDI; DAQ	Costs 3%	NR	Healthcare costs and productivity losses. indirect socioeconomics costs	Societal perspective: €-2,437 [CI NR]	No significant differences between conditions in terms of development of outcome measures over time	Societal perspective: ICER not reported
8 [33]	Patients with somatized mental disorder in Primary care	I: Treatment by GPs having received additional training for somatized mental disorder (103); II: treatment by GPs without additional training (92)	Psychiatric symptom questionnaire (GHQ-12)	NA	1995	Healthcare costs	Healthcare perspective: -€10,464. [CI NR]	Percentage patients no longer GHQ-12 cases: 13% [CI NR]	Healthcare perspective: ICER not reported
9 [32]	Patients who somatize/ patients with 6 to 12 unexplained medical symptoms	I: Patients’ physician receives psychiatric consultation letter (27); II: patients’ physician receive letter after a year (one way cross-over design) (29)	Health outcome measured with RAND Health Status Measures	No	1990	Healthcare costs	Healthcare perspective: -€451 [€62; €724]	Physical functioning: 6.87. General health: -2.23. Mental health: -0.79. Social functioning: -0.97	Healthcare perspective: ICER not reported
10 [31]	Patients with somatization disorder	I: Psychiatric consultation letter (40); II: no psychiatric consultation letter (33)	Mental Health; General Health Rating; Physical Capacity	NA	1990	Healthcare costs	Healthcare perspective: -€710 [-€948. − €386]	Mental Health Index: 5.21 [-0.5; 10.9]. General Health Rating Index: 4.18 [-1.3; 9.6]. Physical Capacity Index: 15.15 [5.4, 24.9]	Healthcare perspective: ICER not reported.
11 [30]	Patients (18–65) with FM recruited from primary health care centres	I: Group ACT (GACT) (51); II: recommended pharmacotherapy (RPT) (52); III: waiting list (53)	QALY (EQ-5D)	NA	2014	Healthcare costs and productivity losses	Healthcare perspective: I vs III; €-1,642 [-2,533; -751]; II vs III; €-745 [-1,751; 261]; I vs II; €-897 [-1,559; -235]. Societal perspective: I vs III €-1,875 [-2,930; -819]; II vs III; €-1,481 [-2,626; -338]; I vs II; €-394 [-1,226; 440].	QALY: I vs III: .05 [.04; .07]; II vs III: .04 [.02; .05]; I vs II: .01 [.00; .03].	Healthcare and societal perspective: I vs III: GACT on average dominant II vs III: RPT on average dominant I vs II: GACT on average dominant
12 [36]	Patients with FM recruited from primary healthcare centres	I: Group-based cognitive behavioural therapy (CBT) (57); II: Recommended pharmacologic treatment (RPT) (56); III: Treatment as usual (TAU) (55)	QALY (EQ-5D)	NA	2011	Healthcare costs, productivity losses	Healthcare perspective: I vs III €-1,748 [-2,938; -558]; I vs II: €-1,931 [-2,983; -879]; II vs III: 183 [-1,110; 1,477]. Societal perspective: I vs III: €-2,311 [-3,593; -1,029]; I vs II: € -2,467 [-3,561; -1,373]; II vs III: 156 [-1,232; 1,544].	QALY: I vs III: 0.02 [-0.00; 0.03]; I vs II: 0.01 [-0.00; 0.03]; II vs III: 0.00 [-0.01; 0.02].	Healthcare perspective: CBT on average dominant vs RPT and TAU. ICER for II vs III equals €105,347 per QALY. Societal perspective: CBT on average dominant vs RPT and TAU. ICER for II vs III equals €84,625 per QALY
13 [58]	Primary care patients meeting the American College of Rheumatology criteria for FM	I: Psychoeducation+usual care (108); II: usual care (108)	QALYs (EQ-5D)	NA	2008	Healthcare costs, productivity losses	Healthcare perspective: -€241 [-690; 323]; Societal perspective: -€221 [-881; 444]	QALY: 0.12 [0.06; 0.19]	Healthcare and societal perspective: the intervention is dominant on average
14 [52]	Patients with FM and men and women with musculoskeletal pain	I: Pregabalin; II: Tramadol/acetaminophen; III: Duloxetine; IV: Gabapentin; V: Amitriptyline; VI: Fluoxetine; VII: Fluoxetine/amitriptyline	Visual Analog Pain Scale Score; Global Improvement (FIQ) of Fibromyalgia	Costs and effects at 5%	2010	Healthcare costs	Healthcare perspective: I vs V: €11,291 [10,559; 12,024]; II vs V: €12,052 [11,175; 12,929]; III vs V: €18,431 [14,996; 21,867]; IV vs V: €14,438 [12,630; 16,246]; VI vs V: €1,063 [865; 1,261]; VII vs V € 1,700 [1,488; 1,911].	Reduction VAS compared to V: I: 22.6% [21%,24%]; II:-4.3% [-5%,-4%]; III: 12.0% [10%,14%];IV: 15.9% [14%,18%]; VI -16.0% [-19%,-13%]; VII: -8.6% [-10%,-8%]. Reduction FIQ compared to V: I: 16.4% [15%,17%]; II: -1.5% [-1.5%,-1.3%]; III: 13.3% [10%,16%]; IV: 13.9% [12%,16%]; VI: -8.6% [-10%,-7%]; VII: 3.6% [3.2%,4%].	Healthcare perspective: For VAS outcomes, V dominated II, VI and VII on average. The other arms had an ICER of 49,906 (arm I), 153,368 (arm III) and 90,623 (arm IV). For FIQ outcomes, V dominated II, VI on average. The other arms had an ICER of 68,850 (arm I), 138,325 (arm III), 103,497 (arm IV) and 46,202 arm (VII).
15 [53]	Patients with severe FM	I: Pregabalin (150 or 225 mg); II: placebo; III: duloxetine (60 or 120 mg; IV: gabapentin; V: tramadol; VI: milnacipran (100 or 200 mg); VII: amitriptyline	Response	NA	NR	Healthcare costs and productivity losses	Societal perspective: pregabalin (150 mg / 225 mg): vs II: €-741 / -1,813; vs III (60mg): €-407 / -1,479; vs III (120mg): €-851 / -1,923; vs IV: €-208 / -1,280; vs V: €490 / -582; vs VI (100 mg): €-762 / -1,834; vs VI (200 mg): €-591 / -1,663; vs VII: €1,029 / -43	Pregabalin (150 mg / 225 mg): vs II: 59.58 / 62.21; vs III (60mg): 28.89 / 31.52; vs III (120mg): 26.64 / 29.27; vs IV: 29.13 / 31.75; vs V: 9.20 / 11.83; vs VI (100 mg): 47.07 / 49.69; vs VI (200 mg): 52.78 / 55.41; vs VII: -10.61 / -7.98	Pregabalin (150 or 225 mg) dominates II, III, IV and VI. Pregabalin 250 mg dominates V, whereas the ICER of pregabalin 250 mg vs V equals €53 per response. Compared to VII, pregabalin 150 mg is being dominated whereas pregabalin 250 mg results in €6 per response.
16 [42]	Patients eligible for pharmacotherapy who had received a clinical diagnosis of FM by fulfilling 1990 ACR classification criteria	I: first-line duloxetine; II: second-line duloxetine, III: guideline-concordant treatment sequence	symptom-control months (SCM); QALY (EQ-5D)	Costs and effects at 3%	2009	Healthcare costs, wider social impacts (e.g., supportive care, home adaptations, and reduced productivity)	Healthcare perspective: I vs III: €548 [CI NR]; II vs III: €136 [CI NR]. Societal perspective: NR	SCM: I vs III: 0.665 [CI NR]. II vs III: 0.460 [CI NR]. QALY: I vs III: 0.0123 [CI NR] II vs III: 0.0087 [CI NR]	Healthcare perspective: I vs III: ICER is €44,754 per QALY; €825 per SCM; II vs III: ICER is €15,587 per QALY; €294 per SCM. Societal perspective: I vs III: ICER is €42,336 per QALY; €781 per SCM; II vs III: ICER is €13,117 per QALY; €247 per SCM
17 [47]	Patients with severe FM, with FM meeting ACR criteria	I: Pregabalin; II: placebo; III: duloxetine; IV: gabapentin; V: tramadol; VI: amitriptyline	Response / QALY (SF-6D)	Costs and effects at 3.5%	2008	Healthcare costs	Healthcare perspective: pregabalin (300 mg / 450 mg): vs II: €891 / 905; vs III (60mg): €377 / 391; vs III (120mg): €252 / 266; vs IV: €719 / 732; vs V: €735 / 749; vs VI: €880 / 895 [CI NR]	Response: pregabalin (300 mg / 450 mg): vs II: 3.40 / 3.55; vs III (60mg): 1.65 / 1.80; vs III (120mg): 1.52 / 1.67 vs IV: 1.66 / 1.81 vs V: 0.53 / 0.68 vs VI: -0.60 / -0.45. QALY: pregabalin (300 mg / 450 mg): vs II: 0.028 / 0.030; vs III (60mg): 0.014 / 0.015; vs III (120mg): 0.013 / 0.014; vs IV: 0.014 / 0.015; vs V: 0.004 / 0.006; vs VI: -0.005 / -0.004.[CI NR]	QALY: I vs II: ICER is €31,416 for 300 mg and €30,558 for 450 mg. I vs III: ICER is below €30,000 for all different doses of pregabalin versus different doses of duloxetine. I vs IV: ICER is €51,834 for 300 mg and €48,464 for 450 mg. I vs V: ICER is €167,787 for 300 mg and €132,999 for 450 mg. I vs VI: Pregabalin (300 and 450 mg) is dominated.
18 [37]	Women with FM according to ACR criteria	I: Aquatic exercise program + usual care (17); II: usual care (16)	QALY (EQ-5D)	NA	2005	Healthcare costs and time and travel costs	Healthcare perspective: €611 [CI NR]. Societal perspective: €1,220 [CI NR].	QALY: 0.131 [0.011; 0.290]	Healthcare perspective: ICER: €4,665 per QALY [2,105; 55,545]Societal perspective: ICER: €9,310 per QALY [4,206; 110,875].
19 [59]	Patients with primary FM according to the ACR 1990 classification criteria	I: Spa treatment (SPA) (58); II: usual care (UC)(76)	QALY (SF-6D)	NA	2000	Healthcare costs, and direct and indirect non-healthcare costs	Societal perspective: €1,894 [-793 to 4,218]	QALY: 0.00 [CI NR]	ICER not reported
20 [60]	Patients meeting the ACR criteria for FM	I: educational discussion group (39); II: Educational cognitive intervention (49); III: Waitlist	QALY^c^	NA	1993	Direct healthcare costs, direct non-healthcare costs, and productivity losses	Societal perspective: €2,303 [CI NR]	QALY: 0.027 [CI NR]	ICER not reported
21 [49]	Adults with moderate to severe IBS with constipation who have previously received antispasmodics and/or laxatives	I: Linaclotide; II: antidepressants	QALY (EQ-5D)	3.5%	2011/2012	Healthcare costs	€809 [CI NR]	QALY: 0.089 [CI NR]	ICER:€9,045 per QALY.
22 [51]	Patients with diarrhoea-predominant or mixed IBS according to Rome III criteria	I: Sacral nerve stimulation (SNS) (26); II: no-treatment (17)	QALY (transformed using GSRS-IBS scores) (EQ-5D)	Costs and effects at 3%	2013	Healthcare costs	Healthcare perspective: €5,949 [4,021; 7,876] at 4-year and €2,897 [2,396; 3,396] at 20-year	QALY: 0.163 [0.146; 0.180] at 4 years; 0.131 [0.1186; 0.1434] at 20 years	ICER: €36,582 per QALY after 4 years; €22,112 per QALY after 20 years.
23 [34]	Adult patients with IBS with constipation	I: Linaclotide; II: Lubiprostone	QALY (EQ-5D) / Response (IBS-QoL)	NA	NR	Healthcare costs and productivity losses	Healthcare perspective: - €88–-€65. Societal perspective: NR	QALY: 0.0004–0.0014. Response: 4.6% - 6.3%	ICER: Linaclotide dominated Lubiprostone on average.
24 [61]	Patients with IBS	I: Acupuncture as adjunct to usual care (116) II: Usual care (117)	QALY (EQ-5D)	NA	2010	Healthcare costs	Healthcare perspective:€291 [-73; 656]	QALY: 0.0035 [-0.0389; 0.0458]	ICER: €83,160 per QALY.
25 [62]	People diagnosed with IBS by a physician and presently fulfilling the ROM III criteria for IBS	I: Internet-based cognitive behaviour therapy (43); II: Internet chat forum (43)	GSRS-IBS	NA	2008	Direct medical costs and direct and indirect non-medical costs	Societal perspective: -€5,437 [CI NR]	Fraction of recovered participants on GSRS-IBS: 0.34 [CI NR]	ICER: €-15,992 (cost saving) per clinically significant improvement
26 [63]	Patients fulfilling the ROME III criteria for IBS	I: Internet-based cognitive behaviour therapy (30); II: waiting list (31)	GSRS-IBS	NA	2010	Direct medical costs and direct and indirect non-medical costs	Societal perspective: -€5,210 [CI NR]	Fraction of recovered participants on GSRS-IBS: 0.14 [CI NR]	ICER: €-37,216 (cost saving) per clinically significant improvement
27 [64]	Patients aged 16–50 years with a clinical diagnosis of IBS	I: CBT + Mebeverine (72); II: Mebeverine (76)	Irritable bowel severity scoring system	NA	2000/2001	healthcare costs and productivity losses	Societal perspective:€515 [CI NR]	Clinically significant change in severity:0.1 [CI NR]	ICER: €5,149 per clinically significant change in IBS severity
28 [35]	Non-diarrhoea IBS patients	I: Tegaserod (247); II: placebo (238)	QALY (EQ-5D)	NA	NR	Treatment costs	Healthcare perspective: €173 [CI NR]	QALY: 0.0077 [CI NR]	ICER:€22,454 per QALY
29 [65]	Patients with IBS 18 years and older	I: Guidebook (141); II: guidebook and self-help group session (139); III: usual care (140)	Patients’ clinical global impression scores	NA	Unclear	Healthcare costs	Healthcare perspective: I and II vs III: -€116 [-€163 to -€68]	Perceived symptom severity: I vs II vs III: 0.51 [0.23; 0.79).	ICER: not reported
30 [50]	Patients fulfilling the ROME II criteria for IBS-D	I: Serologic test for CS followed by endoscopic biopsy for positive tests; II: Empirical IBS treatment	symptomatic improvement	Costs and effects at 3%	NR (clearly)	Healthcare costs	Healthcare perspective: €86 [CI NR]	Symptomatic improvement: -0.07% [CI NR]	ICER: €12,311 per symptomatic improvement.
31 [43]	Patients with severe and very severe symptoms of IBS, at least 6 months and not responding to "usual" medical treatment	I: Psychotherapy (85); II: paroxetine (86); III: treatment as usual (86)	Abdominal pain, SF-36	Costs 6%	1997/1998	Healthcare costs, direct non-health care costs and productivity losses	Healthcare perspective: I vs III: -€591 [CI NR];II vs III: -€404 [CI NR]. Societal perspective: Not reported	SF-36 physical component: I vs III: 5.6 [CI NR]; II vs III: 5.9 {CI NR]. Pain: I vs III: 0.6 [CI NR]; II vs III: -0.7 [CI NR]	ICER: not reported
32 [66]	Patients (18–60) with CFS diagnosed and referred to a rehabilitation centre	I: Multidisciplinary rehabilitation treatment (MRT) (57); II: Cognitive behavioural therapy (CBT) (52)	QALY (EQ-5D), fatigue severity (CIS).	NA	2012	Healthcare costs, patient and family costs, productivity losses	Societal perspective: €5,629 [2,599; 8,452]	QALY: 0.09 [-0.02; 0.19] CIS: -6.48 [-11.54, -1.42]	ICER: €856 per unit of the CIS fatigue subscale. QALY: €118,074 per QALY
33 [67]	Patients aged 18–65 years with at least six months of persistent fatigue	I: Cognitive behavioural therapy based fatigue self-management (FSM) (37); II: usual care (36)	Fatigue Severity Scale (FSS)	NA	2010	Direct healthcare costs; direct non-healthcare costs, indirect costs	Societal perspective: -€1.615 [-4,790 to 1,023]	FSS reduction: 0.73 [0.15; 1.42]	ICER:-€2,203 per FSS gain, indicating that FSS dominates on average.
34 [44]	Patients with CFS/ME diagnosed using the Oxford criteria	I: pragmatic rehabilitation (PR) (95); II: supportive listening (SL) (101); III: treatment as usual (TAU) (100)	QALY (EQ-5D)	Costs and effects at 3.5%	2008/2009	Healthcare costs and productivity losses	Healthcare perspective: I vs III: €289 [€-628; 1,207]; II vs III: €609 [€-331; 1,549].	QALY: I vs III: -0.012 [-0.088; 0.065] II vs III: -0.042 [-0.122; 0.038]	On average, TAU dominates PR and SL
35 [68]	Patients with the Oxford diagnostic criteria for CFS	I: Adaptive Pacing Therapy (APT) (159); II: Cognitive Behaviour Therapy (CBT) (161); III: Graded Exercise Therapy (GET) (160); IV: Specialist Medical Care (SMC) (160)	Chalder Fatigue Scale; SF-36 physical function sub-scale; QALY (EQ-5D)	NA	2009/2010	Healthcare costs and productivity losses	Healthcare perspective: I vs IV: €1,096; II vs IV: €1,204; III vs IV: €1,079. Societal perspective: I vs IV: €2,522; II vs IV: -€930; III vs IV: -€629	QALY: I vs IV: 0.0149; II vs IV: 0.0492; III vs IV: 0.0343. Fatigue: I vs IV: 1.9; II vs IV: 11.1; III vs IV: 14.0. Disability: I vs IV: -8.5; II vs IV: 13.4; III vs IV: 12.6.	ICER: From a healthcare perspective, cost per QALY was €24,475 for CBT, €31,456 for GET and €73,576 for APT. From a societal perspective, CBT and GET dominated SMC on average, whereas SMC was preferred over APT for all outcomes.
36 [38]	Patients from GP practices who had experienced symptoms of fatigue for at least three months	I: Graded-exercise (71); II: counselling (76); III: usual care plus a self-help booklet (75)	Chalder Fatigue Scale	NA	2006/2007	Healthcare costs and social care costs	Healthcare perspective: I vs III: €364 [197; 533]; II vs III: €590 [402; 780];	Chalder improvements: I vs III: 1.1 [-2.3; 4.4]; II vs III: -0.1 [-3.1; 2.9]	ICER: I vs III: €1,377 per unit of clinically significant improvement on the Chalder Fatigue Scale. II vs III: Counselling is dominated by usual care plus self-help booklet
37 [45]	Patients, aged 18–60, with CFS	I: Cognitive behaviour therapy (CBT) (92); II: guided support groups (SG) (90); III: natural course (88)	QALY (EQ-5D), Response	No dis- counting	1998	Healthcare costs and productivity losses	Healthcare perspective: I vs III: €1,440; II vs III: €1,528. Societal perspective: I vs III: -€1,164; II vs III: -€8,519	QALY: I vs III: 0.0279; II vs III: -0.0476. Response: I vs III: 7%; II vs III: -9%.	ICER: I vs III: €28,674 per clinically significant improvement and €29,875 per QALY; I vs II: CBT dominated SG on average
38 [39]	Patients with unexplained fatigue that had lasted for more than 3 months	I: Cognitive behavioural therapy (52); II: graded exercise therapy (50); III: usual care plus a self-help booklet (30)	Chalder fatigue score	NA	2000/2001	Healthcare costs, social services, informal care	I and II combined vs III: €232 [CI NR]	Chalder fatigue score: I and II combined vs III: 4.38	ICER: not reported
39 [40]	Patients from GP practices who had experienced symptoms of fatigue for at least three months	I: Counselling (65); II: cognitive behaviour therapy (64)	Fatigue Questionnaire	NA	1998	Healthcare costs, informal care and productivity losses	Societal perspective: -€316 [-1938; 1,701].	Fatique score: 0.90 [-1.80; 3.60]	ICER: not reported

ACR: American College of Rheumatology; ANTI: anticonvulsant; BDI: Beck Depression Inventory; BID: twice a day; BPI: Brief Pain Inventory; CABAH: Cognitions About Body and Health Questionnaire; CFS: chronic fatigue syndrome; CI: confidence interval; CI NR: confidence interval not reported; CIS: Checklist Individual Strength; CS: celiac sprue; DAQ: Dysfunctional Analysis Questionnaire; DUL: duloxetine; FIQ: Fibromyalgia Impact Questionnaire; FM: fibromyalgia; GHQ-12: 12-item General Health Questionnaire; GSRS-IBS: Gastrointestinal Symptom rating scale-IBS; HRQoL: health related quality of life; IBS: irritable bowel syndrome; ICER: Incremental Cost-Effectiveness Ratio; LOCF: last observation carried forward; MUS: medically unexplained symptoms; NA: not applicable; NR: not reported; PRAM: pramipexole; QALY: quality-adjusted life year; SF-36: Short Form-36; SNRI: Serotonin–norepinephrine reuptake inhibitor; SOMS; Screening for Somatoform Symptoms; TCA: tricyclic antidepressant; TRAM: tramadol; WI: Whiteley Index; WtP: Willingness to Pay.

^a^ Sample size (n) of the intervention conditions applicable only to trial-based economic evaluations.

^b^ Valuation method of utilities applicable only to cost-utility analyses.

^c^ Valuation method unclear.

*Only outcomes as reported in main/base case analyses.

Studies focusing on MUS (*n* = 10) evaluated group training (*n* = 4): a collaborative group intervention [54], cognitive-behavioral group training [46,48] and mindfulness-based cognitive therapy [56]. The other studies [*n* = 6] evaluated individual psychodynamic interpersonal therapy [55], (two-step) cognitive behavioral therapy [41,57], treatment by GPs [33] and a psychiatric consultation letter [31,32]. These interventions were compared with enhanced medical (usual) care [46,54–56], relaxation training [57], waiting list controls [41,48], or no intervention [31–33].

Studies focusing on patients with FM (*n* = 10) compared a pharmacological intervention with another pharmacological intervention (*n* = 4) [42,47,52,53], a group-based therapy (*n* = 2), acceptance and commitment therapy (ACT) [30] and cognitive behavioral therapy (CBT) [36] compared with a pharmacological intervention and treatment as usual or waiting list. An educational intervention (*n* = 2) was compared with usual care or an educational discussion group [58,60] and an aquatic exercise program (*n* = 1) [37] or spa treatment (*n* = 1) [59] was compared with usual care.

In studies focusing on IBS (*n* = 11), a pharmacological intervention (*n* = 3) was compared with another pharmacological intervention or placebo [34,35,49], and internet-based cognitive behavior therapy (*n* = 3) was compared with an internet chat forum, waiting list or pharmacological intervention [62–64] and other studies (*n* = 3) compared sacral nerve stimulation [51], acupuncture [61], or a guidebook [65] with usual care. Psychotherapy (*n* = 1) was compared with a pharmacological intervention [43] and celiac sprue testing (*n* = 1) was compared with empirical therapy [50].

In studies focusing on CFS (*n* = 8), a cognitive behavioral therapy (*n* = 6) [39,40,45,67,68] was compared with usual care, adaptive pacing therapy, graded exercise therapy, specialist medical care or counselling. Graded-exercise (*n* = 1) was compared with counseling or usual care [38]. Pragmatic rehabilitation or multidisciplinary rehabilitation treatment (*n* = 2) was compared with supportive listening, treatment as usual or CBT [44,66].

#### Effects, costs and uncertainty

Information on effect measurement and valuation is described in Table 2. Seventeen studies included only a cost-effectiveness analysis (CEA), fifteen studies only a cost-utility analysis (CUA), seven studies included both a CEA and a CUA. In studies with CEAs, outcomes were expressed as costs per unit improvement on a (clinical) outcome measure. In studies with CUAs, outcomes were expressed as costs per QALY gained, where the majority of the CUAs (*n* = 14) elicited utilities using the EuroQol 5D (EQ-5D).

TBEE studies (*n* = 30) included healthcare costs, patient and family costs including productivity losses (*n* = 15) [30,36,40,41,43–46,54,56,58,60,64,66,68]; direct treatment costs (*n* = 2) [35,55], i.e. costs that are directly related to the intervention being studied; healthcare costs (*n* = 6) [31–33,57,61,65] or intervention costs or work related costs and healthcare costs (*n* = 7) [37–39,59,62,63,67].

Almost all TBEE studies described the method of measuring costs; in two studies [41,46] it was unclear how costs were measured. In 13 TBEEs [31–33,36,37,41,55,57,58,60,62,63,67] it was not clearly reported how costs were valued. In 13 TBEEs [30,35–37,40,43,44,54,55,58,61,62,66] uncertainty was handled by means of bootstrapping and additional sensitivity analyses. In 11 studies [38,39,45,46,56,59,60,63,64,67,68] bootstrapping without additional sensitivity analyses or sensitivity analyses without bootstrapping were performed. In the remaining six TBEEs [31–33,41,57,65] neither a bootstrapping procedure nor additional sensitivity analyses were performed.

MBEE studies (n = 9) [34,42,47–53] included healthcare costs, patient and family costs including productivity losses (*n* = 2) [34,53]; healthcare costs and work related costs (*n* = 2) [42,48] and healthcare costs (*n* = 5) [47,49–52] The cost sources were reported in seven MBEEs [34,47–50,52,53]. Both probabilistic and deterministic sensitivity analyses were conducted in six MBEEs [34,48–52], whereas in three MBEEs [42,47,53] either a probabilistic or a deterministic sensitivity analysis was conducted.

### Quality assessment

Tables 3 and 4 describe the CHEC quality scores per item. None of the included studies met all CHEC criteria. Two items, discussing the generalizability of the results (item 18) and ethical and distributional issues (item 20), had the lowest scores. Three studies discussed the generalizability of the study results properly [43,47,50], whereas in the remaining studies the generalizability of results only was mentioned but not discussed (*n* = 10) [30,36,37,41,46,54,58,63,66,67] or not described at all (*n* = 26). On average, the items on well-defined research question (item 3), appropriateness of the economic study designs (item 4) and identified important and relevant outcomes (item 11) had the highest scores. One study [65] defined the research question incomplete, for all 39 studies the economic study design was considered appropriate.

**Table 3:** Quality assessment CHEC-extended.

Item	Study ID																				
	1	2	3	4	5	6	7	8	9	10	11	12	13	14	15	16	17	18	19	20	21	
1.Is the study population clearly described?	0.5	0.5	1	0.5	0.5	1	1	1	1	0.5	1	1	1	0	0.5	0.5	1	1	1	0.5	0.5	
2.Are competing alternatives clearly described?	1	1	1	1	1	1	1	1	1	0	1	1	1	0.5	0.5	0.5	0.5	1	1	1	0.5	
3.Is a well-defined research question posed in answerable form?	1	1	1	1	1	1	1	1	1	1	1	1	1	1	1	1	1	1	1	1	1	
4.Is the economic study design appropriate to the stated objective?	1	1	1	1	1	1	1	1	1	1	1	1	1	1	1	1	1	1	1	1	1	
5.Are the structural assumptions and the validation methods of the model properly reported (models only)?	NA	NA	0	NA	NA	NA	NA	NA	NA	NA	NA	NA	NA	0.5	0.5	1	0.5	NA	NA	NA	0	
6.Is the chosen time horizon appropriate in order to include relevant costs and consequences?	1	1	1	1	1	1	1	0	1	1	0.5	0.5	1	1	1	1	1	0.5	1	1	1	
7.Is the actual perspective chosen appropriate?	1	1	1	0.5	1	0	1	0	0	0	1	1	1	0.5	1	1	0.5	1	1	1	0.5	
8.Are all important and relevant costs for each alternative identified?	1	1	1	0	1	0.5	1	1	1	1	1	1	1	1	1	0	0.5	0.5	1	1	1	
9.Are all costs measured appropriately in physical units?	0	1	1	1	1	1	0	1	1	1	1	1	1	1	0	1	1	1	1	1	1	
10.Are costs valued appropriately?	0.5	0.5	1	0	1	0	0	0	0	0	0.5	0	0	0	0	0	X	0	1	0	1	
11.Are all important and relevant outcomes for each alternative identified?	1	1	1	1	1	1	1	1	0.5	1	1	1	1	1	1	1	1	1	1	1	1	
12.Are all outcomes measured appropriately?	1	1	1	1	1	1	1	1	1	1	1	1	1	1	1	1	1	1	1	1	1	
13.Are outcomes valued appropriately?	1	1	1	1	1	NA	NA	NA	NA	NA	1	1	1	NA	NA	1	1	1	1	0	1	
14.Is an appropriate incremental analysis of costs and outcomes of alternatives performed?	1	1	1	1	1	0	0	1	0	0	1	1	1	1	1	1	1	1	1	0	1	
15.Are all future costs and outcomes discounted appropriately?	0	NA	1	NA	NA	NA	0.5	NA	0	NA	NA	NA	NA	1	NA	1	1	NA	NA	NA	1	
16.Are all important variables, whose values are uncertain, appropriately subjected to sensitivity analysis?	0.5	1	1	1	0.5	0	0	0	0	0	1	1	1	1	0.5	0.5	0.5	1	0.5	0.5	1	
17.Do the conclusions follow from the data reported?	1	1	0.5	0.5	1	1	1	0.5	1	1	1	0.5	1	0.5	0.5	0.5	0	1	0.5	1	1	
18.Does the study discuss the generalizability of the results to other settings and patient/client groups?	0.5	0.5	0	0	0	0	0.5	0	0	0	0.5	0.5	0.5	0	0	0	1	0.5	0	0	0	
19.Does the article/ report indicate that there is no potential conflict of interest of study researcher(s) and funder(s)?	1	1	0.5	1	1	1	0	0	0	1	1	1	1	0	1	1	1	1	1	0	1	
20.Are ethical and distributional issues discussed appropriately?	0	0	0	0	0	0	0	0	0	0	0	0	0	0	0	0	0	0	0	0	0	

0: no, 0.5: suboptimal, 1: yes, X: unclear, NA: not applicable

**Table 4:** Quality assessment CHEC-extended (studies 22–39) continued.

Item	Study ID																	
	22	23	24	25	26	27	28	29	30	31	32	33	34	35	36	37	38	39	
1.Is the study population clearly described?	0.5	0	0	1	1	1	0	1	1	1	1	1	0.5	0.5	0.5	1	0.5	0.5	
2.Are competing alternatives clearly described?	1	0.5	0.5	1	1	1	0.5	1	0.5	1	1	1	1	1	1	1	1	0	
3.Is a well-defined research question posed in answerable form?	1	1	1	1	1	1	1	0.5	1	1	1	1	1	1	1	1	1	1	
4.Is the economic study design appropriate to the stated objective?	1	1	1	1	1	1	1	1	1	1	1	1	1	1	1	1	1	1	
5.Are the structural assumptions and the validation methods of the model properly reported (models only)?	0	0.5	NA	NA	NA	NA	NA	NA	1	NA	NA	NA	NA	NA	NA	NA	NA	NA	
6.Is the chosen time horizon appropriate in order to include relevant costs and consequences?	1	0	1	1	1	1	0	1	1	1	1	1	1	1	0.5	1	0.5	0.5	
7.Is the actual perspective chosen appropriate?	0	1	0.5	1	1	1	0	0.5	0	0.5	1	1	0.5	1	0.5	1	0.5	1	
8.Are all important and relevant costs for each alternative identified?	0	1	1	1	1	1	0	1	1	1	1	1	1	1	1	1	1	1	
9.Are all costs measured appropriately in physical units?	1	1	1	1	1	1	1	1	0	1	1	1	1	1	1	1	1	1	
10.Are costs valued appropriately?	0.5	0	1	0	0	1	1	0.5	0	0.5	1	0	1	1	1	1	1	1	
11.Are all important and relevant outcomes for each alternative identified?	1	1	1	1	1	1	1	1	1	1	1	1	1	1	1	1	1	1	
12.Are all outcomes measured appropriately?	0	0.5	1	1	1	1	1	1	1	1	1	1	1	1	1	1	1	1	
13.Are outcomes valued appropriately?	1	1	1	NA	NA	NA	1	NA	NA	NA	1	NA	1	1	NA	1	NA	NA	
14.Is an appropriate incremental analysis of costs and outcomes of alternatives performed?	1	0	1	1	1	1	1	0	1	0	1	1	1	1	1	1	1	0	
15.Are all future costs and outcomes discounted appropriately?	1	NA	NA	NA	NA	NA	NA	NA	1	0.5	NA	NA	1	NA	NA	0	NA	NA	
16.Are all important variables, whose values are uncertain, appropriately subjected to sensitivity analysis?	1	1	1	1	0.5	0.5	1	0	1	1	1	0.5	1	0.5	0.5	0.5	0.5	1	
17.Do the conclusions follow from the data reported?	0.5	1	1	1	1	0	1	1	0.5	1	1	1	1	1	1	1	1	1	
18.Does the study discuss the generalizability of the results to other settings and patient/client groups?	0	0	0	0	0.5	0	0	0	1	1	0.5	0.5	0	0	0	0	0	0	
19.Does the article/ report indicate that there is no potential conflict of interest of study researcher(s) and funder(s)?	1	0.5	0.5	1	1	1	0.5	1	0	0.5	1	1	1	1	1	0	0	0	
20.Are ethical and distributional issues discussed appropriately?	0	0	0	0	0	0	0	0	0	0	0	0	0	0	0	0	0	0	

0: no, 0.5: suboptimal, 1: yes, X: unclear, NA: not applicable.

### Outcomes

The incremental costs, incremental effects and health economic results in terms of ICERs of the reference-cases are presented in Table 2. Twenty-two (five of which on MUS [46,48,54,55,56]; eight on FM [30,36,37,42,47,58,59,60], five on IBS [34,35,49,51,61], four on CFS [44,45,66,68]) out of 39 studies included a CUA with QALYs as outcome. ICERs were reported in 24 studies and in 13 of these studies interventions [30,34,36,37,42,46,47,48,49,51,54,58,68] were dominant over the control condition. Group interventions focusing on MUS (*n* = 3)[46,48,54] or FM (*n* = 4) [30,36,37,58] might be more cost-effective in comparison with individual interventions. Four pharmacological interventions focusing on FM (*n* = 2) [42,47] or IBS (*n* = 2) [34,49] and two individual interventions focusing on IBS (*n* = 1) [51] or CFS (*n* = 1) [68] appeared to be cost-effective in comparison with the control condition.

#### Medically unexplained symptoms

In studies focusing on MUS, four studies assessed the cost-effectiveness of group interventions [46,48,54,56] and one study an individual intervention [55]. The group interventions, group CBT program (STreSS) [46], collaborative group intervention [54], and cognitive-behavioural group training [48] appeared to be cost-effective, but the mindfulness-based cognitive group therapy [56] was not cost-effective. The individually administered psychodynamic interpersonal therapy [55] was not cost-effective either. Each of these interventions was compared to enhanced usual care or enhanced medical care, except for the use of a wait-list condition in Visser et al.[48].

#### Fibromyalgia

In the studies focusing on FM, the aquatic exercise program [37] appeared to be cost-effective whereas spa treatment [59] was not cost-effective; both group interventions were compared to usual care. Three other studies on group-based cognitive behavioral therapy (CBT) [36], additional psychoeducation[58] or group based acceptance and commitment therapy (ACT) [30] were cost-effective compared to recommended pharmacologic treatment or treatment as usual or waiting list or usual care. In an older study [60] educational cognitive intervention was not cost-effective in comparison with an educational discussion group. In two studies, both funded by the pharmaceutical industry, a pharmacological intervention was compared with another pharmacological intervention. Duloxetine as second-line treatment was cost-effective [42] and pregabalin 450 mg appeared to be cost-effective in comparison with duloxetine 120 mg, but pregabalin 450 mg was not cost-effective compared to tramadol, amitriptyline or placebo [47].

#### Irritable bowel syndrome

Three studies focused on IBS [34,35,49], all funded by the pharmaceutical industry, compared a pharmacological intervention with another pharmacological intervention or placebo. Linaclotide appeared to be more cost-effective in comparison with antidepressants [49] and with lubiprostone [34]. Tegaserod [35] did not appear to be more cost-effective than placebo. Sacral nerve stimulation, a non-pharmacological intervention [51], appeared to be cost-effective in comparison with no treatment. Another non-pharmacological intervention, acupuncture as adjunct to usual care [61], was not cost-effective in comparison with usual care.

#### Chronic fatigue syndrome

In studies including patients with CFS, the interventions pragmatic rehabilitation and supportive listening appeared to be not cost-effective compared to treatment as usual [44]. On average, individual cognitive behavior therapy (CBT) was more cost-effective in comparison with specialist medical care [68]. In another study [45] CBT was compared with guided support groups (SG) and the natural course. CBT was less costly and more effective than SG and even cost-effective in comparison with the natural course of the disease. Compared to CBT, multidisciplinary rehabilitation treatment [66] appeared not to be cost-effective.

## Discussion

### Main findings

To our knowledge, this is the first systematic review of cost-effectiveness of interventions for undifferentiated MUS and the three most well-known functional syndromes FM, IBS and CFS with a methodological quality assessment of the included studies. We identified 39 full economic evaluations of interventions for treating patients with MUS and FSS. Heterogeneity of the included studies concerning interventions, time horizon, and outcome was high. Twenty-two out of 39 studies included a CUA with QALYs as outcome. In 13 CUAs the intervention conditions dominated the control conditions or had an ICER below the WTP threshold of € 50,000 per QALY, meaning that the interventions were (on average) cost-effective in comparison with the control conditions. In nine CUAs the intervention condition was not cost-effective compared with the control conditions. Group interventions focusing on MUS (*n* = 3) or FM (*n* = 4) might be more cost-effective than individual interventions.

### Discussion of the results

Although this study provides valuable information regarding existing evidence on the cost-effectiveness of interventions for MUS, the comparability of the studies included in this systematic review was limited due to heterogeneity in terms of interventions, (economic) study design, time horizon and outcome measures. The variety in effect measures used in the economic evaluations limits the comparability of the studies and their results. Only twenty-two studies used both clinical effects and QALYs as outcome. The other studies included CEAs using diagnosis-specific measures. The variety in outcome measures amongst these studies was high.

A general limitation of the included studies is that the costs of somatic specialist care were often not taken into account, while it can be expected that proper treatment of MUS can lead to a decrease in these costs. This could potentially result in underestimation of the cost-effectiveness of the interventions.

The studies included in this systematic review contained four studies [31–33,41] that were also part of an earlier systematic review on the cost-of-illness and economic evaluations of interventions for MUS disorders by Konnopka et al.[18]. In that systematic review, 13 studies were included with patients with MUS, five cost-of-illness studies and eight economic evaluations, of which only two cost-effective analyses. Similar to our review the comparability of included studies was limited due to the heterogeneity concerning design, methods and year of study conduct.

An intervention may be cost-effective only after a longer period of time. One study [48] did show through a modeling approach that cognitive behavioral group intervention could be cost-effective after 21 months. The time horizon of only 12 months used in many studies may be the reason that interventions were not cost-effective. An important advantage of MBEEs is that they allow cost-effectiveness to be modelled over longer periods, although at the cost of more uncertainty. Six out of nine included MBEEs had a time horizon of at least two years.

#### Quality assessment

Due to the variability of the study methodological quality, drawing conclusions regarding the cost-effectiveness of different types of interventions focusing on different target populations is difficult. Assessing the methodological quality of the studies using the CHEC-list [23], a validated checklist for the methodological quality assessment of economic evaluations, is partly subjective and we have limited ourselves to discuss all items separately. To enhance the reliability we applied the procedure as mentioned in the methods paragraph. Moreover, the separate items of the CHEC-list were valued equally and the overall quality score therefore does not reflect that certain items, such as the chosen time horizon and the chosen perspective, could be perceived as having a relatively large impact on study methodological quality. In the included studies the chosen time horizon was usually appropriate and the chosen perspective depended on the healthcare system of the country of the study.

### Strengths and limitations

This review has several strengths. We used a broad search strategy in which psychological, medical and health economic literature databases were searched thoroughly. We included all health economic studies focusing on interventions for MUS with the three most prevalent FSS. Furthermore, the quality of the included studies was appraised with the CHEC [23]. Additionally, we applied the recommended strategy for conducting and reporting systematic reviews [19]. In this review we only presented base case results and not the results of sensitivity analyses. Therefore decision makers should consider context-specific factors (e.g. cost-reimbursement of interventions) when deciding on implementing interventions.

This study also has some limitations. The literature search had language restrictions (only English, German, and Dutch), but we assume that economic evaluations will be published mainly in international journals. The included patient groups vary in terms of reported severity, i.e. number and duration of symptoms, functional disability or quality of life. This might constitute a limitation in terms of how MUS are defined but also whether the patients have similar characteristics at inclusion in terms of, e.g. length of illness before treatment, severity of symptoms or previous treatments. Cost-effectiveness of different interventions is not only supposed to depend on the intervention itself, but also on underlying medical (and demographic) conditions and prognostic factors such as number of symptoms, number of body systems involved and number of times symptoms are presented. [10].

With regard to the interventions, it should be noted that the availability of the pharmacotherapy and non-pharmacological interventions can differ per country.

Another limitation is the methodological quality assessment with the CHEC. The CHEC list [23] is the best available instrument for the quality assessment of economic evaluations, and is recommended by the Cochrane Handbook for Systematic Reviews of Interventions [24] for critical appraisal of the methodological quality of health-economic evidence. The presented descriptive information and quality assessment gives a broad picture of the study designs and study quality, but the information is incomplete and decision makers should look in detail at the studies of interest, especially so for the model-based economic evaluations, for which the CHEC-list is less appropriate as a quality assessment tool.

As all included studies were conducted in Western countries, the generalizability of the results is presumably limited due to differences in healthcare provision of a country.

### Recommendations

This systematic review provides an overview of group interventions and individual interventions for patients with MUS. Further research is needed to investigate the willingness to participate in group interventions. To address the disease burden and societal costs associated with MUS, it is important to know the cost-effectiveness of available interventions. While the current review shows that not all interventions are cost-effective, this could also be the result of choices made in the included studies such as a relatively short time horizon and the chosen perspective. Due to the chronic nature of MUS and the societal costs MUS may cause outside of the healthcare domain (e.g. lost productivity), it is recommended to conduct high quality economic evaluations of interventions for patients with MUS, with a long time horizon and a chosen perspective in line with the national or local guidelines and the decision makers’ information requirements. The studies would ideally use both perspectives, healthcare and societal perspective, so that the outcomes become more relevant for decision makers in different settings. High quality economic evaluations are necessary in order to draw robust conclusions about the cost-effectiveness of interventions for MUS.

## Conclusion

The current review provides an overview of 39 studies of interventions for patients with MUS, FM, IBS and CFS and the methodological quality assessment of these studies. In 13 out of 22 studies the intervention condition dominated the control conditions, meaning that the interventions were (on average) cost-effective in comparison with the control conditions. Considering the limited comparability due to the heterogeneity of the studies, the group interventions might be more cost-effective than individual interventions.

## Supporting information

S1 Table PRISMA checklist. (DOCX)

S2 Table Studies excluded on full-text level. (DOCX)

S1 Text Protocol of the study, PROSPERO. (PDF)

S1 Appendix Search strings. (DOCX)

S2 Appendix CHEC extended scoring instruction. (DOCX)
