# Supplementary material for: Cost-effectiveness of interventions for medically unexplained symptoms: A systematic review
Source: PLoS One. 2018 Oct 15;13(10):e0205278. doi: 10.1371/journal.pone.0205278 (PMC6188754; doi:10.1371/journal.pone.0205278)
Supplement: S2 Table — (DOCX) [file pone.0205278.s002.docx]

| Appendix 4. Table: Studies excluded on full-text level | | | | | | |
| --- | --- | --- | --- | --- | --- | --- |
| **Authors (year)** | **Title and reference** | **Country** | **Target Population** | **Excluded for reasons** | | |
|  |  |  |  | **No full Economic evaluation** | **No original Research** | **No MUS** |
| van Ravesteijn et al., 2016 | Mindfulness-based cognitive therapy for patients with somatoform disorders. Tijdschr Psychiatr 2016; 58(3):198-206. | Netherlands | MUS |  | x |  |
| Fjorback et al., 2013 | Mindfulness therapy for somatization disorder and functional somatic syndromes: analysis of economic consequences alongside a randomized trial. J Psychosom Res 2013; 74(1):41-48. |  | MUS | x |  |  |
| Konnopka et al., 2012 | Economic s of medically unexplained symptoms: a systematic review of the literature. Psychother Psychosom 2012; 81(5):265-275. | German | MUS |  | x |  |
| Schade et al., 2011 | Cost-efficiency of a brief family intervention for somatoform patients in primary care. Fam Syst Health 2011; 29(3):197-205. |  | MUS | x |  |  |
| Luo et al., 2007 | Costs of an intervention for primary care patients with medically unexplained symptoms: a randomized controlled trial. Psychiatr Serv 2007; 58(8):1079-1086. |  | MUS | x |  |  |
| Timmer et al., 2006 | Die verhaltenstherapeutische Behandlung von Patienten mit somatoformen Störungen im Rahmen der stationären Rehabilitation. Verhaltenstherapie und Verhaltensmedizin. = Behavioral Therapy for patients with somatoform disorders within inpatient rehabilitation. Verhaltenstherapie & Verhaltensmedizin 2006; 27(3):363-381. | German | MUS | x |  |  |
| Larisch et al., 2005 | Kosten-Nutzen-Aspekte psychosozialer Interventionen bei somatisierenden Patienten in der Hausarztpraxis. = cost-effectiveness of psychosocial interventions for somatising patients by the general practitioner. Zeitschrift f++r Klinische Psychologie und Psychotherapie: Forschung und Praxis 2005; 34(4):282-290. | German | MUS | x |  |  |
| Hiller et al., 2004 | Effects and **COST**-effectiveness analysis of inpatient treatment for somatoform disorders. Fortschr Neurol Psychiatr 2004; 72(3):136-146. |  | MUS | x |  |  |
| Ryan et al., 2004 | Biofeedback-based psychophysiological treatment in a primary care setting: an initial feasibility study. Appl Psychophysiol Biofeedback 2004; 29(2):79-93. |  | MUS | x |  |  |
| Albrecht et al., 2000 | Concept for a health care economic evaluation of short- and long-term costs and effectiveness parameters of an expanded ambulatory psychosomatic rehabilitation program. Gesundheitswesen 2000; 62(3):156-160. | German | MUS |  |  | x |
| Bhusal et al., 2016 | Clinical utility, safety, and efficacy of pregabalin in the treatment of fibromyalgia. Drug Healthc Patient Saf 2016; 8:13-23 |  | FM |  | x |  |
| Feliu-Soler et al., 2016 | Cost-utility and biological underpinnings of Mindfulness-Based Stress Reduction (MBSR) versus a psychoeducational programme (FibroQoL) for fibromyalgia: a 12-month randomised controlled trial (EUDAIMON study). BMC Complement Altern Med 2016; 16(1):81. |  | FM |  | x |  |
| Parker et al., 2015 | A systematic review of pharmacoeconomic studies for pregabalin. Pain Pract 2015; 15(1):82-94 |  | FM |  | x |  |
| Skaer et al., 2014 | Fibromyalgia: disease synopsis, medication **COST** effectiveness and economic burden. Pharmaco economics 2014; 32(5):457-466. |  | FM | x |  |  |
| Keshavarz et al., 2013 | A systematic cost-effectiveness analysis of pregabalin in the management of fibromyalgia: an Iranian experience. Arch Med Sci 2013; 9(6):961-967. |  | FM |  | x |  |
| Kleinman et al., 2011 | Health outcomes and costs among employees with fibromyalgia treated with pregabalin vs. standard of care. Pain Pract 2011; 11(6):540-551. |  | FM | x |  |  |
| Robinson et al., 2006 | In search of pharmacoeconomic evaluations for fibromyalgia treatments: a review. Expert Opin Pharmacother 2006; 7(8):1027-1039. |  | FM |  | x |  |
| Buesing et al., 2005 | A conservative, cost-effective approach to fibromyalgia. JAAPA 2005; 18(9):32-37. |  | FM | x |  |  |
| Maetzel et al., 1998 | A review of cost-effectiveness analyses in rheumatology and related disciplines. Curr Opin Rheumatol 1998; 10(2):136-140. |  | FM |  | x |  |
| McCain et al., 1996 | A cost-effective approach to the diagnosis and treatment of fibromyalgia. Rheum Dis Clin North Am 1996; 22(2):323-349. |  | FM | x |  |  |
| Lightfoot et al., 1993 | Empiric parenteral antibiotic treatment of patients with fibromyalgia and fatigue and a positive serologic result for Lyme disease. A cost-effectiveness analysis. Ann Intern Med 1993; 119(6):503-509. |  | FM |  |  | x |
| Rogers et al., 1989 | Pain clinic #14. Fibromyalgia and myofascial pain: either, neither, or both? Orthop Rev 1989; 18(11):1217-1224. |  | FM | x |  |  |
| [Sun et al.,](https://www.ncbi.nlm.nih.gov/pubmed/?term=Sun%20P%5BAuthor%5D&cauthor=true&cauthor_uid=23489659) 2014 | [Wu A](https://www.ncbi.nlm.nih.gov/pubmed/?term=Wu%20A%5BAuthor%5D&cauthor=true&cauthor_uid=23489659) Direct medical costs and medication compliance among fibromyalgia patients: duloxetine initiators vs. pregabalin initiators.[Pain Pract.](https://www.ncbi.nlm.nih.gov/pubmed/?term=Direct+Medical+Costs+and+Medication+Compliance+among+Fibromyalgia+Patients) 2014 Jan;14(1):22-31. |  | FM | x |  |  |
| Flik et al., 2015 | Comparison of medical costs generated by IBS patients in primary and secondary care in the Netherlands. BMC Gastroenterol 2015; 15:168. |  | IBS | x |  |  |
| Mohseninejad et al., 2013 | Targeted screening for Coeliac Disease among irritable bowel syndrome patients: analysis of cost-effectiveness and value of information. Eur J Health Econ 2013; 14(6):947-957 |  | IBS | x |  |  |
| Lynd et al., 2010 | Using the incremental net benefit framework for quantitative benefit-risk analysis in regulatory decision-making-a case study of alosetron in irritable bowel syndrome. Value Health 2010; 13(4):411-417. |  | IBS | x |  |  |
| Creed et al., 2008 | The relationship between somatisation and outcome in patients with severe irritable bowel syndrome. J Psychosom Res 2008; 64(6):613-620. |  | IBS | x |  |  |
| Creed et al., 2005 | Outcome in severe irritable bowel syndrome with and without accompanying depressive, panic and neurasthenic disorders. The British Journal of Psychiatry 2005; 186(6):507-515. |  | IBS | x |  |  |
| Gilkin et al., 2005 | The spectrum of irritable bowel syndrome: A clinical review. Clin Ther 2005; 27(11):1696-1709. |  | IBS |  | x |  |
| Smith et al., 2005 | Tegaserod treatment for IBS: a model of indirect costs. Am J Manag Care 2005; 11(1 Suppl):S43-S50. |  | IBS | x |  |  |
| Costa et al., 2001 | A cognitive group therapy program for irritable bowel syndrome: A comprehensive interdisciplinary model. US: ProQuest Information & Learning, 2001. |  | IBS | x |  |  |
| Martin et al., 2001 | Irritable bowel syndrome: toward a cost-effective management approach. Am J Manag Care 2001; 7(8 Suppl):S268-S275. |  | IBS |  | x |  |
| Harris et al., 1997 | Irritable bowel syndrome. A cost-effective approach for primary care physicians. Postgrad Med 1997; 101(3):215-20, 223. |  | IBS | x |  |  |
| Collin et al., 2011 | The impact of CFS/ME on employment and productivity in the UK: a cross-sectional study based on the CFS/ME national outcomes database. BMC Health Serv Res 2011; 11:217. |  | CFS | x |  |  |
| van Dam et al., 2011 | Kosten, kosteneffectiviteit en implementatie van getrapte zorg voor het chronisch vermoeidheidssyndroom. = costs, cost-effectiveness and implementation of stepped-care for chronic fatigue syndrome. Gedragstherapie 2011; 44(3):191-205. | Netherlands | CFS | x |  |  |
| O'Dowd et al., 2006 | Cognitive behavioural therapy in chronic fatigue syndrome: a randomised controlled trial of an outpatient group programme. Health Technol Assess 2006; 10(37):iii-x, 1. |  | CFS | x |  |  |
| Stouten et al., 2004 | Cost-effectiveness of cognitive behaviour therapy for patients with chronic fatigue syndrome. QJM 2004; 97(6):379-380. | Netherlands | CFS |  | x |  |
| Scheeres et al., 2008 | Implementing cognitive behavior therapy for chronic fatigue syndrome in mental health care: a costs and outcomes analysis., *BMC Health Serv Res*,2008; 8():175 |  | CFS | x |  |  |
| Richardson et al., 2008 | Cost effectiveness of the Expert Patients Programme (EPP) for patients with chronic conditions Epidemiol Community Health 2008;62:361–367. doi:10.1136/jech.2006.057430 |  | CFS |  |  | x |
