# Supplementary material for: Cost-effectiveness of interventions for medically unexplained symptoms: A systematic review
Source: PLoS One. 2018 Oct 15;13(10):e0205278. doi: 10.1371/journal.pone.0205278 (PMC6188754; doi:10.1371/journal.pone.0205278)
Supplement: S1 Text — (PDF) [file pone.0205278.s003.pdf]

PROSPERO International prospective register of systematic reviews

Review title and timescale

1 Review title

Give the working title of the review. This must be in English. Ideally it should state succinctly the interventions or exposures being reviewed and the associated health or social problem being addressed in the review.

Cost-effectiveness of interventions for patients with medically unexplained symptoms: a systematic review

2 Original language title

For reviews in languages other than English, this field should be used to enter the title in the language of the review. This will be displayed together with the English language title.

3 Anticipated or actual start date

Give the date when the systematic review commenced, or is expected to commence.

01/02/2017

4 Anticipated completion date

Give the date by which the review is expected to be completed.

01/10/2017

5 Stage of review at time of this submission

Indicate the stage of progress of the review by ticking the relevant boxes. Reviews that have progressed beyond the point of completing data extraction at the time of initial registration are not eligible for inclusion in PROSPERO. This field should be updated when any amendments are made to a published record.

The review has not yet started ×

Review stage	Started	Completed
Preliminary searches	Yes	No
Piloting of the study selection process	Yes	No
Formal screening of search results against eligibility criteria	No	No
Data extraction	No	No
Risk of bias (quality) assessment	No	No
Data analysis	No	No

Provide any other relevant information about the stage of the review here.

Review team details

6 Named contact

The named contact acts as the guarantor for the accuracy of the information presented in the register record.

Margreet Wortman

7 Named contact email

Enter the electronic mail address of the named contact.

m.s.h.wortman@hva.nl

8 Named contact address

Enter the full postal address for the named contact.

Tafelbergweg 51, 1105 BD Amsterdam, The Netherlands

9 Named contact phone number

Enter the telephone number for the named contact, including international dialing code.

0031621156825

10 Organisational affiliation of the review

Full title of the organisational affiliations for this review, and website address if available. This field may be completed as 'None' if the review is not affiliated to any organisation.

ACHIEVE-Centre of Applied Research, Faculty of Health, Amsterdam University of Applied Sciences, Amsterdam,

The Netherlands and Department of General Practice and Elderly Care Medicine, Amsterdam Public Health Research Institute, VU University Medical Center, Amsterdam, The Netherlands

Website address:
http://www.hva.nl/achieve and https://www.amsterdamresearch.org/web/public-health/home.htm

11 Review team members and their organisational affiliations
Give the title, first name and last name of all members of the team working directly on the review. Give the organisational affiliations of each member of the review team.

Title	First name	Last name	Affiliation
Ms	Margreet	Wortman	ACHIEVE-Centre of Applied Research, Faculty of Health, Amsterdam University of Applied Sciences, Amsterdam, The Netherlands; Department of General Practice and Elderly Care Medicine, Amsterdam Public Health Research Institute, VU University Medical Center, Amsterdam, The Netherlands
Dr	Joran	Lokkerbol	Trimbos Institute Utrecht, The Netherlands; Rob Giel Research Center, University Medical Center Groningen, Groningen, The Netherlands
Dr	Tim	olde Hartman	Department of Primary and Community Care, Radboud University Medical Center, Nijmegen, The Netherlands
Dr	Bart	Visser	ACHIEVE-Centre of Applied Research, Faculty of Health, Amsterdam University of Applied Sciences, Amsterdam, The Netherlands
Professor	Willem	Assendelft	Department of Primary and Community Care, Radboud University Medical Center, Nijmegen, The Netherlands
Professor	Henriëtte	van der Horst	Department of General Practice and Elderly Care Medicine, Amsterdam Public Health Research Institute, VU University Medical Center, Amsterdam, The Netherlands

12 Funding sources/sponsors
Give details of the individuals, organizations, groups or other legal entities who take responsibility for initiating, managing, sponsoring and/or financing the review. Any unique identification numbers assigned to the review by the individuals or bodies listed should be included.
Netwerk Kwaliteitsontwikkeling GGZ P140018 t.b.v. Zorgstandaard Somatisch Onvoldoende verklaarde Lichamelijke Klachten (SOLK)

13 Conflicts of interest
List any conditions that could lead to actual or perceived undue influence on judgements concerning the main topic investigated in the review.
Are there any actual or potential conflicts of interest?
None known

14 Collaborators
Give the name, affiliation and role of any individuals or organisations who are working on the review but who are not listed as review team members.

Title	First name	Last name	Organisation details

Review methods

15 Review question(s)

State the question(s) to be addressed / review objectives. Please complete a separate box for each question.
The overall aim of this review is to identify and present an overview of published full economic evaluations of interventions for patients with medically unexplained symptoms (MUS). The interventions that will be included are: psychological interventions, pharmacological interventions, physical therapies, eHealth interventions, and blended interventions. Furthermore, the quality of the identified studies will be assessed.

What is the evidence regarding cost-effectiveness of interventions for medically unexplained symptoms?

What is the methodologicial quality of the identified economic evaluations of interventions for medically unexplained symptoms?

16 Searches
Give details of the sources to be searched, and any restrictions (e.g. language or publication period). The full search strategy is not required, but may be supplied as a link or attachment.
The following databases will be searched: PubMed, PsycINFO, National Health Service Economic Evaluations Database (NHS EED) and the Cost-Effectiveness Analysis (CEA) registry. We used Medical Subject Headings (MeSH) terms and Psychological Index Terms for searches within the PubMed and PsycINFO databases respectively. Free text-words and controlled vocabulary will be combined with predefined searches for economic evaluations. Filters for economic evaluations providing maximal sensitivity will be used. (Glanville et al., 2009) Furthermore, reference lists of eligible economic evaluations will be searched. The search will be limited to publications in the English, Dutch, and German languages.

17 URL to search strategy
If you have one, give the link to your search strategy here. Alternatively you can e-mail this to PROSPERO and we will store and link to it.

I give permission for this file to be made publicly available
Yes

18 Condition or domain being studied
Give a short description of the disease, condition or healthcare domain being studied. This could include health and wellbeing outcomes.
Interventions for MUS. The term ‘MUS’ is used to cover a wide range of symptoms which cannot be clearly explained by a general medical condition, even after a thorough examination and any relevant investigations. Key feature of symptom-defined conditions such as fibromyalgia, irritable bowel syndrome and chronic fatigue syndrome are persistent somatic symptoms that are not sufficiently explained by structural or otherwise specified pathology after a thorough physical examination (Henningsen et al., 2007). Patients presenting with MUS may vary in terms of reported severity i.e. number of symptoms, functional disability or quality of life, and duration of symptoms.

19 Participants/population
Give summary criteria for the participants or populations being studied by the review. The preferred format includes details of both inclusion and exclusion criteria.
Patients diagnosed with medically unexplained symptoms or function somatic symptoms. Diagnosis of MUS may be either by validated instrument (e.g. PHQ-15) or clinician judgement. Patients with functional somatic symptoms (FSS) will be included, e.g. irritable bowel syndrome (IBS), chronic fatigue syndrome (CFS), fibromyalgia. Population should include adults aged 18 years or over. Depending on the search results, subgroups will be distinguished.

20 Intervention(s), exposure(s)
Give full and clear descriptions of the nature of the interventions or the exposures to be reviewed
All interventions for medically unexplained symptoms will be included: psychological interventions, pharmacological interventions, physical therapies, eHealth interventions and blended interventions.

21 Comparator(s)/control
Where relevant, give details of the alternatives against which the main subject/topic of the review will be compared (e.g. another intervention or a non-exposed control group).
There will be no restrictions on interventions in the control conditions. It is expected that the majority of control conditions will be care-as-usual, psychological treatment, pharmacological treatment, or waiting list.

22 Types of study to be included
Give details of the study designs to be included in the review. If there are no restrictions on the types of study design

eligible for inclusion, this should be stated.
Full economic evaluations (either trial-based or model-based) comparing at least 2 different interventions in terms of cost effectiveness (including cost-utility) for medically unexplained symptoms will be included. Exclusion criteria: - Studies with interventions focusing on prevention; - Costs of illness studies; - Studies focusing on occupational health setting; - Studies with medically (partly) explained symptoms or medically unexplained symptoms as secondary diagnosis; - Articles that are not original studies (systematic reviews etc.).

23 Context
Give summary details of the setting and other relevant characteristics which help define the inclusion or exclusion criteria.
Patients with MUS, all populations and settings.

24 Primary outcome(s)
Give the most important outcomes.
Incremental cost-utility ratios, cost-effectiveness ratios, cost-benefit ratios, and cost-consequence ratios.

Give information on timing and effect measures, as appropriate.

25 Secondary outcomes
List any additional outcomes that will be addressed. If there are no secondary outcomes enter None.
Methodological quality.

Give information on timing and effect measures, as appropriate.

26 Data extraction (selection and coding)
Give the procedure for selecting studies for the review and extracting data, including the number of researchers involved and how discrepancies will be resolved. List the data to be extracted.
After deletion of duplicate studies, title and abstract of each retrieved study will be independently screened by two review authors to identify studies that potentially meet the inclusion criteria outlined above. The full text of these potentially eligible studies will be independently assessed for eligibility by two review team members. Any disagreement between them over the eligibility of particular studies will be resolved through discussion with a third reviewer, at the level of the titles/abstract screening as well as of full text screening. Inclusion will be based on consensus. After exclusion of non-eligible studies, data from the included studies will be retrieved for analysis.

27 Risk of bias (quality) assessment
State whether and how risk of bias will be assessed, how the quality of individual studies will be assessed, and whether and how this will influence the planned synthesis.
The quality of the included economic evaluations will be assessed using the Consensus on Health Economics Checklist (CHEC) (Evers et al., 2005). Quality assessment of each included study will be performed by at least two reviewers. The final quality assessment will be based on consensus.

28 Strategy for data synthesis
Give the planned general approach to be used, for example whether the data to be used will be aggregate or at the level of individual participants, and whether a quantitative or narrative (descriptive) synthesis is planned. Where appropriate a brief outline of analytic approach should be given.
Depending on the data of the included economic evaluations, outcomes will be aggregated. Data analysis will be descriptive if the heterogeneity is high. Incremental ratios will be reported and transformed into Euros (€).

29 Analysis of subgroups or subsets
Give any planned exploration of subgroups or subsets within the review. 'None planned' is a valid response if no subgroup analyses are planned.
Subgroups will be distinguished depending on the included studies.

Review general information

30 Type and method of review
Select the type of review and the review method from the drop down list.
Systematic review

Public health (including social determinants of health)

31 Language
Select the language(s) in which the review is being written and will be made available, from the drop down list. Use the control key to select more than one language.
English

Will a summary/abstract be made available in English?
Yes

32 Country
Select the country in which the review is being carried out from the drop down list. For multi-national collaborations select all the countries involved. Use the control key to select more than one country.
Netherlands

33 Other registration details
Give the name of any organisation where the systematic review title or protocol is registered together with any unique identification number assigned. If extracted data will be stored and made available through a repository such as the Systematic Review Data Repository (SRDR), details and a link should be included here.

34 Reference and/or URL for published protocol
Give the citation for the published protocol, if there is one.
Give the link to the published protocol, if there is one. This may be to an external site or to a protocol deposited with CRD in pdf format.

I give permission for this file to be made publicly available
Yes

35 Dissemination plans
Give brief details of plans for communicating essential messages from the review to the appropriate audiences.
The systematic review will be published in a peer-reviewed international scientific journal and disseminated at local and international conferences.

Do you intend to publish the review on completion?
Yes

36 Keywords
Give words or phrases that best describe the review. (One word per box, create a new box for each term)
Cost-effectiveness

Interventions

Medically unexplained symptoms

Treatment outcome

Cost-Benefit Analysis

37 Details of any existing review of the same topic by the same authors
Give details of earlier versions of the systematic review if an update of an existing review is being registered, including full bibliographic reference if possible.

38 Current review status
Review status should be updated when the review is completed and when it is published.
Ongoing

39 Any additional information

Provide any further information the review team consider relevant to the registration of the review.

40 Details of final report/publication(s)

This field should be left empty until details of the completed review are available.
Give the full citation for the final report or publication of the systematic review.
Give the URL where available.
