# Supplementary material for: Cost-effectiveness of interventions for medically unexplained symptoms: A systematic review
Source: PLoS One. 2018 Oct 15;13(10):e0205278. doi: 10.1371/journal.pone.0205278 (PMC6188754; doi:10.1371/journal.pone.0205278)
Supplement: S1 Appendix — (DOCX) [file pone.0205278.s004.docx]

**S2 Appendix Search strings.**

1. **PubMed**

#6. Search (therapy OR therapies OR therapeutic OR treating OR treatment OR treatments psychotherapy OR psychotherapeutic OR psychotherapeutical OR psychotherapeutics OR intervention OR interventions OR pharmacotherapy OR pharmacotherapeutic OR psychopharmacotherapy OR psychopharmacotherapeutic*)

#7. Search (“medically unexplained symptoms” OR “unexplained symptoms” OR “medically unexplained” OR “unexplained disease” OR “unexplained diseases” OR “unexplained illness” OR “unexplained illnesses” OR “unexplained physical symptoms” OR “unexplained medical symptoms” OR “unexplained health symptoms” OR “unexplained somatic symptoms” OR “unexplained somatic complaints” OR “unexplained physical complaints” OR frequent attend* OR high utili* OR somatization OR somatisation OR hypochondri* OR non specific complaint* OR nonspecific complaint* OR psychogen*[tw] OR non specific sympt* OR nonspecific sympt* OR functional somatic syndrom* OR functional somatic sympt* OR functional syndrom* OR“functional disorder*”[ti] OR “functional disease*”[ti] OR “Somatoform Disorders”[Mesh] OR “Psychosomatic Medicine”[Mesh] OR “Psychophysiologic Disorders”[Mesh] OR somatoform disorder* OR psychosomatic medicine OR psychosomatic disorder OR psychophysiologic disorder* OR “unexplained complaints” OR “subjective health complaints” OR “nonspecific chest pain” OR “non specific chest pain” OR fibromyalgia OR “irritable bowel syndrome” OR “chronic fatigue” OR “fatigue syndrome” OR “non cardiac pain” OR “noncardiac pain” OR “non cardiac chest pain” OR “noncardiac chest pain” OR “Chest Pain/psychology”[Mesh:noexp] OR “Tension-Type Headache”[Mesh] OR tension headache* OR “non specific headache” OR “nonspecific headache” OR trichotillomania OR neurastheni* OR “post viral fatigue” OR scratching[ti] OR “psychogenic excoriation” OR “neurotic excoriation” OR “compulsive skin picking” OR dermatotillomania OR “acne excoriee” OR “Burning Mouth Syndrome”[Majr] OR “Tinnitus”[Majr] OR “Whiplash Injuries”[Mesh] OR dysmorphophobia OR “body dysmorphic” OR “phantom pain” OR “Phantom Limb”[Mesh] OR conversion disorder* OR repetitive strain injury OR “repetitive strain injury” OR “sick building syndrome” OR “multiple chemical sensitivity syndrome” OR “temporomandibular dysfunction” OR “myalgic encephalitis” OR “post viral fatigue” OR “chronic pelvic pain syndrome” OR “post whiplash syndrome” OR “chronic low back pain” OR “functional dyspepsia” OR “premenstrual syndrome” OR “chronic benign pain syndrome” OR “Gulf Syndrome” OR “pelvic pain” OR “interstitial cystitis” OR “neck pain” OR “back pain” OR “Fatigue”[Mesh] OR “dizziness”[MeSH])

#8. Search (#6 AND #7)

#12. Search (("cost effectiveness"[tiab] OR "cost utility"[tiab] OR "economic evaluation"[tiab] OR “economic evaluations”[tiab] OR "health economic model"[tiab] OR “health economic models”[tiab] OR "return on investment"[tiab] OR “return on investments”[tiab] OR "costs and benefits"[tiab] OR "cost benefit data"[tiab] OR "discrete event simulation" [tiab]))

#13. Search "Models, Economic"[Mesh] OR "Costs and Cost Analysis"[Mesh:noexp] OR "Cost-Benefit Analysis"[Mesh] OR "Markov Chains"[Mesh] OR "Decision Trees"[Mesh]

#14. Search (#12 OR #13)

#15. Search (#8 AND #14)

#16. Search "Cost of Illness"[Mesh] OR "Health Care Costs"[Mesh]

#17. Search (#14 OR #16)

#18. Search (#8 AND #17)

#19. Search (#18 NOT #15)

Limiters - Language: English, Dutch, German

1. **PsycINFO via Interface – EBSCOhost**

S1. TI/KW/AB

S2. ( “medically unexplained symptoms” OR “unexplained symptoms” OR “medically unexplained” OR “unexplained disease” OR “unexplained diseases” OR “unexplained illness” OR “unexplained illnesses” OR “unexplained physical symptoms” OR “unexplained medical symptoms” OR “unexplained health symptoms” OR “unexplained somatic symptoms” OR “unexplained somatic complaints” OR “unexplained physical complaints” OR frequent attend* OR high utili* OR somatization OR somatisation OR hypochondri* OR non specific complaint* OR nonspecific complaint* OR psychogen* OR non specific sympt* OR nonspecific sympt* OR functional somatic syndrom* OR functional somatic sympt* OR functional syndrom* OR “functional disorder*” OR “functional disease*” OR “Somatoform Disorders” OR “Psychosomatic Medicine” OR “Psychophysiologic Disorders” OR somatoform disorder* OR psychosomatic medicine OR psychosomatic disorder OR psychophysiologic disorder* OR “unexplained complaints” OR “subjective health complaints” OR “nonspecific chest pain” OR “non specific chest pain” OR fibromyalgia OR “irritable bowel syndrome” OR “chronic fatigue” OR “fatigue syndrome” OR “non cardiac pain” OR “noncardiac pain” OR “non cardiac chest pain” OR “noncardiac chest pain” OR “Chest Pain” OR “Tension-Type Headache” OR tension headache* OR “non specific headache” OR “nonspecific headache” OR trichotillomania OR neurastheni* OR “post viral fatigue” OR scratching OR “psychogenic excoriation” OR “neurotic excoriation” OR “compulsive skin picking” OR dermatotillomania OR “acne excoriee” OR “Burning Mouth Syndrome” OR “Tinnitus”OR “Whiplash” OR dysmorphophobia OR “body dysmorphic” OR “phantom pain” OR “Phantom Limb” OR conversion disorder* OR “repetitive strain injury” OR “sick building syndrome” OR “multiple chemical sensitivity syndrome” OR “temporomandibular dysfunction” OR “myalgic encephalitis” OR “post viral fatigue” OR “chronic pelvic pain syndrome” OR “post whiplash syndrome” OR “chronic low back pain” OR “functional dyspepsia” OR “premenstrual syndrome” OR “chronic benign pain syndrome” OR “Gulf Syndrome” OR “pelvic pain” OR “interstitial cystitis” OR “neck pain” OR “back pain” OR Fatigue OR dizziness )

S3. DE "Somatoform Disorders" OR DE "Body Dysmorphic Disorder" OR DE "Conversion Disorder" OR DE "Hypochondriasis" OR DE "Neurasthenia" OR DE "Neurodermatitis" OR DE "Somatization Disorder" OR DE "Somatoform Pain Disorder" or DE "Conversion Disorder" OR DE "Hysterical Paralysis" OR DE "Hysterical Vision Disturbances" OR DE "Pseudocyesis" or DE "Psychosomatic Medicine" OR DE "Scratching" or DE "Whiplash"

S4. S2 OR S3

S5. psychotherapeutical OR psychotherapeutics OR intervention OR interventions OR pharmacotherapy OR pharmacotherapeutic OR psychopharmacotherapy OR psychopharmacotherapeutic* ) OR KW ( therapy OR therapies OR therapeutic OR treating OR treatment OR treatments psychotherapy OR psychotherapeutic OR psychotherapeutical OR psychotherapeutics OR intervention OR interventions OR pharmacotherapy OR pharmacotherapeutic OR psychopharmacotherapy OR psychopharmacotherapeutic* ) TI ( therapy OR therapies OR therapeutic OR treating OR treatment OR treatments psychotherapy OR psychotherapeutic OR psychotherapeutical OR psychotherapeutics OR intervention OR interventions OR pharmacotherapy OR pharmacotherapeutic OR psychopharmacotherapy OR psychopharmacotherapeutic* ) OR AB ( therapy OR therapies OR therapeutic OR treating OR treatment OR treatments psychotherapy OR psychotherapeutic OR

S6. S4 AND S5

S7. DE "Costs and Cost Analysis" OR DE "Health Care Economics" OR DE "Markov Chains"

S8. TI ( "discrete event simulation" OR "decision tree" OR "costs and benefits" OR "cost benefit data" OR "cost effectiveness" OR "cost utility" OR "economic evaluation*" OR "health economic model*" OR "return on investment*" ) OR AB ( "discrete event simulation" OR "decision tree" OR "costs and benefits" OR "cost benefit data" OR "cost effectiveness" OR "cost utility" OR "economic evaluation*" OR "health economic model*" OR "return on investment*" ) OR KW ( "discrete event simulation" OR "decision tree" OR "costs and benefits" OR "cost benefit data" OR "cost effectiveness" OR "cost utility" OR "economic evaluation*" OR "health economic model*" OR "return on investment*" )

S9. S7 OR S8

S10. S6 AND S9

S11. DE "Health Care Costs"

S12. S9 OR S11

S13. S6 AND S12

S14. S13 NOT S10

Limiters - Language: English, Dutch, German

1. **NHS-EED via CRD**
2. Somatoform disorders OR somatisation disorder OR undifferentiated somatoform disorder OR somatoform disorder unspecified OR somatoform autonomic dysfunction OR pain disorder OR MUS OR Fibromyalgia OR chronic fatigue syndrome OR irritable bowel syndrome
3. **CEA-registry**
4. Somatoform disorders OR somatisation disorder OR undifferentiated somatoform disorder OR somatoform disorder unspecified OR somatoform autonomic dysfunction OR pain disorder OR MUS OR Fibromyalgia OR chronic fatigue syndrome OR irritable bowel syndrome
