# Supplementary material for: Cost-effectiveness of interventions for medically unexplained symptoms: A systematic review
Source: PLoS One. 2018 Oct 15;13(10):e0205278. doi: 10.1371/journal.pone.0205278 (PMC6188754; doi:10.1371/journal.pone.0205278)
Supplement: S2 Appendix — (DOCX) [file pone.0205278.s005.docx]

## S3 Appendix CHEC extended scoring instruction [22]

Developed for the research project “Title: Cost-Effectiveness of Interventions for Medically Unexplained Symptoms: A Systematic Review”. This instruction applies to the extended version of the Consensus on Health Economic Criteria (CHEC) checklist [21].

**Scoring options:**

0 = No

0,5 = Suboptimal

1 = Yes

NA = Not applicable

X = Unclear

- - TBEE: trial-based economic evaluation
  - MBEE: model-based economic evaluation
  - Only score ‘Unclear’ when none of the other options is appropriate

**General remark:** when authors refer to another article for certain information (for example inclusion and exclusion criteria), score **0,5** when it is clear what specific information is described in another article, and **0** when it is not clear what specific information is described in another article. Only data from the included articles will be checked, not referred articles.

- - **CHEC instruction**

1. **Is the study population clearly described?**

***TBEE:*** Are the clinical characteristics, eligibility, entry and follow-up mentioned? Clinical characteristics including eligibility, entry and follow-up described: **1**

Clinical characteristics, eligibility, entry and follow-up not completely described: **0,5**

No description: **0**

***MBEE:*** Are the clinical characteristics of the (hypothetical) population described? Completely described: **1**

Not clearly described: **0,5**

Not described: **0**

1. **Are competing alternatives clearly described?**

Are the alternative interventions described completely, including duration/intensity? All aspects completely described: **1**

Mentioned but not clearly described: **0,5**

Neither mentioned nor described: **0**

1. **Is a well-defined research question posed in answerable form?**

Is a clear question/objective described? Are the population and interventions included in the research question/objective? This does not have to be a literal question; clear aims or objectives are also sufficient.

Clear research question: **1**

Incomplete research question: **0,5**

No research questioned mentioned: **0**

1. **Is the economic study design appropriate to the stated objective?**

Are both costs and effects of 2 or more interventions compared?

A score of **1** must be assigned; otherwise the study is excluded from the review.

1. **Are the structural assumptions and the validation methods of the model properly reported? (MBEE only)**

Is the structure of the model clearly described and/or depicted, and are the assumptions explained properly?

Model structure is clear and assumptions discussed: **1**

Only model structure or assumptions discussed: **0,5**

Not reported: **0**

1. **Is the chosen time horizon appropriate in order to include relevant costs and consequences?**

Time horizon not mentioned: **0**

Societal perspective study: a time horizon <1 year is short. Time horizon 1 year or beyond: **1**

Time horizon 6 months - 1 year: **0,5**

Time horizon < 6 months: **0**

Healthcare perspective: a time horizon <1 year is common, but it depends on the context whether it is appropriate.

1. **Is the actual perspective chosen appropriate?**

**This item is only for the mentioning of the perspective, not the actual perspective!**

When the perspective is narrower than societal, a justification must be provided.

Societal: **1**

Perspective not explicitly mentioned: **0**

Other/Perspective narrower than societal, but not explained: **0,5**

1. **Are all important and relevant costs for each alternative identified?**

Healthcare perspective

Only intervention costs: **0** Intervention costs and other healthcare costs: **1**

Societal perspective

Only intervention costs: **0**

Intervention costs and healthcare costs: **0,5**

Intervention costs and healthcare costs + costs beyond healthcare: **1**

1. **Are all costs measured appropriately in physical units?**

***TBEE:***

Is it clear how costs are measured? Interview, questionnaire etc.

***MBEE:***

Are the sources of cost data clear and appropriate? Mentioned and appropriate: **1**

Mentioned but inappropriate: **0,5**

Not mentioned: **0**

1. **Are costs valued appropriately?**

Are the sources of valuation including the reference year mentioned for ALL cost categories? Sources and reference/index year mentioned: **1**

Sources described but no reference/index year: **0,5**

Sources not mentioned: **0**

**Note:**

When tariffs or extrapolation are used the score is never 1. Valuation partly based on tariffs or extrapolation: **0,5**

Only tariffs used for all cost categories: **0**

1. **Are all important and relevant outcomes for each alternative identified?**

Are the outcomes relevant and do they fit the research question and perspective?

Outcomes identified and relevant: **1**

Outcomes not identified/not relevant: **0**

1. **Are all outcomes measured appropriately?**

***TBEE:***

Is the measurement instrument relevant and described?

***MBEE:***

Are the sources of the outcome parameters in the model clear and appropriate?

Relevant and described: **1**

Described but not relevant: **0,5**

Not clear/mentioned: **0**

1. **Are outcomes valued appropriately? (CUA/CBA only)**

How are utilities derived (EQ-5D, SF-36, VAS, TTO, etc.)?

Valuation mentioned and appropriate: **1**

Valuation mentioned but not appropriate: **0,5**

Not mentioned: **0**

1. **Is an appropriate incremental analysis of costs and outcomes of alternatives performed**?

Is an ICER calculated? (C2-C1/O2-O1) Only a description (e.g. intervention A has more effects at lower costs compared to B) is not sufficient.

Incremental analysis (C2-C1/O2-O1) performed: **1**

Only (C2-C1) and (O2-O1) performed, but not divided by each other: **0,5**

No incremental analysis of costs and outcomes: **0**

1. **Are all future costs and outcomes discounted appropriately?**

Only for studies > 1 year time horizon.

< 1 year time horizon: **NA**

> 1 year time horizon: Not discounted: **0**

Discount rates are reported for both cost and effects (QALYs): **1**

Discount rates only mentioned for costs or effects: **0,5**

1. **Are all important variables, whose values are uncertain, appropriately subjected to sensitivity analysis?**

***MBEE:***

Probabilistic sensitivity analysis and deterministic sensitivity analysis: **1**

Only probabilistic or deterministic sensitivity analysis: **0,5**

None: **0**

***TBEE:***

Sample uncertainty (bootstrapping) and additional sensitivity analysis performed: **1**

Bootstrapping (sampling uncertainty) without additional sensitivity analysis: **0,5**

Sensitivity analysis without bootstrapping: **0,5**

No bootstrapping or additional sensitivity analysis: **0**

1. **Do the conclusions follow from the data reported?**

Are the results interpreted correctly and cautiously, and are the conclusions supported by the data? For example, when it is concluded that a certain intervention is cost-effective only based on a point- estimate (without bootstrapping and CEAC etc.) this is regarded inappropriate.

Appropriate conclusion: **1**

No conclusion or inappropriate conclusion: **0**

1. **Does the study discuss the generalizability of the results to other settings and patient/client groups?**

Is it addressed how results can vary in other populations, regions, settings?

Mentioned and discussed: **1**

Mentioned: **0,5**

None: **0**

1. **Does the article/ report indicate that there is no potential conflict of interest of study researcher(s) and funder(s)?**

A conflict of interest statement does not have to be literal, when it is stated that authors receive grants for example, this is also sufficient.

When conflicts of interest and/or funding roles are disclosed FOR ALL AUTHORS: **1**

When authors do have competing interests or receive grants etc., but it is clearly disclosed for all authors: **1**

Not mentioned: score **0**

Not clearly or partly disclosed: **0,5**

1. **Are ethical and distributional issues discussed appropriately?**

Are ethical aspects regarding characteristics of the population or interventions discussed? Mentioned and discussed: **1**

Mentioned: **0,5**

None: **0**
